# The topology of metabolic isotope labeling networks

**DOI:** 10.1186/1471-2105-8-315

**Published:** 2007-08-29

**Authors:** Michael Weitzel, Wolfgang Wiechert, Katharina Nöh

**Affiliations:** 1 Department of Simulation, University of Siegen, 57068 Siegen, Germany; 2 Institute of Biotechnology, Research Centre Jülich, 52425 Jülich, Germany

## Abstract

**Background:**

Metabolic Flux Analysis (MFA) based on isotope labeling experiments (ILEs) is a widely established tool for determining fluxes in metabolic pathways. Isotope labeling networks (ILNs) contain all essential information required to describe the flow of labeled material in an ILE. Whereas recent experimental progress paves the way for high-throughput MFA, large network investigations and exact statistical methods, these developments are still limited by the poor performance of computational routines used for the evaluation and design of ILEs. In this context, the global analysis of ILN topology turns out to be a clue for realizing large speedup factors in all required computational procedures.

**Results:**

With a strong focus on the speedup of algorithms the topology of ILNs is investigated using graph theoretic concepts and algorithms. A rigorous determination of all cyclic and isomorphic subnetworks, accompanied by the global analysis of ILN connectivity is performed. Particularly, it is proven that ILNs always brake up into a large number of small strongly connected components (SCCs) and, moreover, there are natural isomorphisms between many of these SCCs. All presented techniques are universal, i.e. they do not require special assumptions on the network structure, bidirectionality of fluxes, measurement configuration, or label input. The general results are exemplified with a practically relevant metabolic network which describes the central metabolism of *E. coli *comprising 10390 isotopomer pools.

**Conclusion:**

Exploiting the topological features of ILNs leads to a significant speedup of all universal algorithms for ILE evaluation. It is proven in theory and exemplified with the *E. coli *example that a speedup factor of about 1000 compared to standard algorithms is achieved. This widely opens the door for new high performance algorithms suitable for high throughput applications and large ILNs. Moreover, for the first time the global topological analysis of ILNs allows to comprehensively describe and understand the general patterns of label flow in complex networks. This is an invaluable tool for the structural design of new experiments and the interpretation of measured data.

## Background

### Metabolic Flux Analysis

Metabolic fluxes essentially represent the *phenotype *of cellular function and regulation. Accordingly, the determination and analysis of cellular flux distributions is a central concern of Systems Biology. Restricting to steady state analysis, available methods contributing to the determination of fluxes within metabolic networks can be roughly divided into two mainstreams which are both based on the stoichiometry.

The first type, Metabolic Network Analysis (MNA), involves tools suitable for exploring the solution space of the stoichiometric equations for comprehensive models. Typically, topological properties of genome-scale networks are under investigation in order to derive information about the set of all feasible flux patterns that would emerge under predefined assumptions. Extreme Pathway Analysis [1,2], Elementary Flux Mode Analysis [3], and Flux Balance Analysis [4] are among the most prominent approaches in this field assessing the theoretical capabilities of metabolic networks. The second type is Metabolic Flux Analysis (MFA), which, in contrast to MNA, is concerned with the quantitative determination of metabolic fluxes in a concrete cell under in-vivo conditions. Supplementing stoichiometry with experimental data produces precisely one such flux distribution.

Isotope labeling networks (ILNs) are the structural backbone of MFA [5-7] and the tracing of isotopic labeling became the dominating method for the determination of fluxes in the central metabolic pathways of microorganisms and higher cells in-vivo [8-11]. The knowledge of these fluxes under a variety of growth conditions or genetic modifications of an organism constitutes one important building block in the *Omics *data family called the *Fluxome *[12].

In contrast to MNA, the networks subject to MFA are typically confined to the central metabolic pathways of an organism and the surrounding biosynthesis pathways of particular interest. For example, the *E. coli *network discussed in this contribution (cf. Fig. 5) contains all amino acid production pathways and, thus, is already a rather large representative network for typical MFA applications. The difference in size between genome-scale networks and metabolic networks suitable for MFA studies is currently due to limited knowledge about atom transitions outside the biosynthesis on the one hand, and by problems with the measurement techniques when low concentrated metabolites are to be analyzed on the other hand.

The centerpiece of all computational MFA routines is the determination of the emerging labeling distribution (see also *Appendix A*). This simulation step is the essential operation for all further steps in the course of experimental evaluation – i.e. parameter fitting, statistical analysis, or experimental design [13-15]. Several computational tools are available for MFA which facilitate the evaluation of isotopic labeling data generated by NMR and MS instruments [16-18]. Although the algorithms underlying these tools are different, basically all of them rely on the structure of the ILN associated with a metabolic network.

#### Current challenges

The recent years brought up several new experimental techniques and requirements resulting in increasing performance demands for MFA tools:

• High-throughput MFA procedures with hundreds of ^13^C labeling experiments running in parallel are now possible and will be certainly used frequently in the future, in the field of Systems Biology [19].

• When MFA is used in screening investigations, detecting the presence or absence of certain metabolic pathways is an important issue and requires a fully automated evaluation of different network variants in a sequence of data evaluation runs (model selection) [20].

• More and more complex metabolic networks are now being studied including not only the central metabolism but also the biosynthesis pathways or the compartmentation in higher cells [21,22]. The availability of elaborate analytics facilitates the use of novel substrates which provide isotopic labelings also in non-carbon atoms (such as H, O, N, etc.) [23]. Thus, the dimension of isotope labeling systems dramatically increases.

• The computation of confidence regions for the estimated fluxes based on nonlinear statistical methods is usually based on Monte-Carlo simulations for which a large number of simulation runs have to be performed [24,25]. In particular, this way, the usually badly determined exchange fluxes of bidirectional reactions can be quantified more reliably [26].

• Whereas the classical ILE is based on a measurement from the isotopically stationary phase of an experiment, isotopically instationary methods are now possible due to the rapid development of MS technology [27]. These experiments require a change from stationary isotopomer balance equations (IBEs) to dynamic differential equation systems. This in turn increases the computational complexity of the problem by several orders of magnitude [28].

Altogether, the computational requirements for these new techniques emphasize the importance of the development of new high performance algorithms for the solution of the arising simulation problems.

#### Attempts to overcome the performance bottleneck of MFA algorithms

Several strategies for solving labeling equation systems have been discussed in the literature [29-32]. However, they all suffer from poor performance, or even do not converge in any case. Most of them have a computational complexity of O(*n*^3^) where *n *is the number of labeled species in the system, i.e. isotopomers, which typically are much higher than 1000.

Having this fact in mind, the current computational bottleneck of isotopic MFA can be illustrated by the following consideration: Assume that the simulation of label distribution for given flux values usually takes a few seconds on a current PC, e.g. for the large *E. coli *network discussed in this contribution. Then, flux estimation is accomplished by an iterative parameter fitting procedure (cf. *Appendix A*, Fig. 10).

Depending on the problem, several hundred simulation steps have to be performed for a single fit. Clearly, each parameter fit should be recurred some times to avoid local optima. For high-throughput MFA the whole procedure has to be repeated for each of possibly several hundreds of samples [12]. When finally the network size grows by some factor > 4 (leading to a factor of about 250 in computational time), the total time for performing a high-throughput MFA study ends up at a couple of weeks which is unacceptable.

Several attempts have been made to speed up the central computational steps for MFA. Most of them rely on an analysis of the specific network for the biological system under investigation and thus, cannot be generalized to an arbitrary network structure. All these methods are based on special assumptions on the reversibility of fluxes [33], path tracing in bidirectional computation steps [34], computer algebraic solution of subsystems [35-37], or the derivation of special relations between measured labels and flux ratios [38]. However, none of these methods is comprehensively applicable.

An universally applicable framework for solving the simulation task which is able to deal with any given network structure and any type of unidirectional or bidirectional fluxes is the cumomer method [14]. Using the graph-based methods described in this contribution a speedup of factor 10^3 ^has been achieved for the presented *E. coli *network. Recently, the elementary metabolite unit (EMU) approach for modeling isotope labeling systems has been proposed in [39] which is specially suited for systems with many labeled species (such as C, H, O, N, etc.). Relying on the back-tracing of measured mass isotopomer spectra to precursors in the central metabolism a special flux analysis algorithm has been developed that reduces the dimension of the forward simulation problem compared to the cumomer concept. Hence, depending on the given measured data set the computation time can be reduced considerably. Interestingly, both approaches – cumomers, and EMUs describing the flow of labeled material in a metabolic network – share the same network structure. Restricting ourselves on cumomers, nevertheless, all methods derived in the present contribution should be profitable for the EMU approach, too.

In this contribution a major effort was taken to translate the structure and terminology of ILEs to graph theoretical terminology. Hence, it is assumed that the reader of this contribution is familiar with elementary graph theory and its formalism. The reader should be familiar with the notion of algorithmic complexity. Moreover, this text should not be considered as a tutorial for isotope-based MFA. A short introduction into the general procedure of isotopic MFA can be found in *Appendix A*. For more details the reader is referred to more comprehensive texts like [5-7].

### Labeling networks and associated balance equations

#### An illustrative example

**Figure 1:**
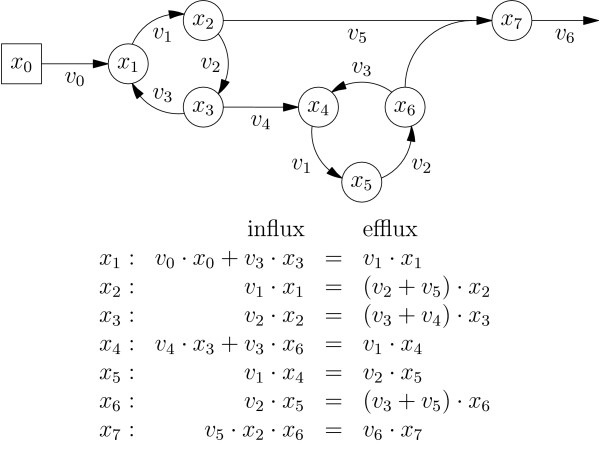
**Illustrative example network**. The network's cyclic subsystems given by node sets {*x*_1_, *x*_2_, *x*_3_} and {*x*_4_, *x*_5_, *x*_6_} share the same fluxes form algebraic subsystems, as well. The second cycle depends on the first. Node *x*_7 _depends on nodes *x*_2 _and *x*_6 _by a quadratic term.

A broad variety of approaches for the description of the flow of labeled material through a metabolic network exist in the literature. These approaches generally differ in the representation of the labeling state. Examples include carbon atoms [40,41], isotopomers [26,41], cumomers [14,42], bondomers [43,44], and EMUs [39]. Basically, all of these representations result in a similar structure of network graphs composed of labeled compounds (the nodes), and metabolic fluxes (the edges) Figure 1.

An illustrative example shall be used to explain the general strategy for improving the performance of MFA algorithms as proposed by this contribution. Fig. 1 shows a section of a general ILN along with the corresponding set of labeling balance equations (LBEs) which quantitatively describe the distribution of labeled material over the network. For the comprehension of the subsequent sections the following properties of LBEs are of importance:

1. For each node *x *of an ILN exactly one equation is formulated, which relates node *x *with its direct neighbors.

2. The balance equation for node *x *always contains *x *and its coefficient is the sum of its label effluxes.

3. Additionally, the balance equation associated with node *x *contains the labeling fractions corresponding to all *upstream *neighbors feeding node *x *with labeled material.

4. In contrast, the *downstream *nodes fed by *x *do not contribute to the balance equation of *x*.

5. The balance equations contain nonlinear terms when *hyper-edges *with multiple sources *w*_1_... *w*_*n *_and a single target *x *occur in the network, i.e. in the case when larger molecules are assembled of smaller molecules.

6. The ILN and the corresponding system of LBEs contain the same information, meaning that each one can be constructed from the other.

#### Cyclic interdependencies

These properties establish the correspondence between networks and equation systems which is well known from the theory of general equation solving [45,46] where the structural network representation of an equation system is known as a computational graph. In particular, it has been shown that the performance of equation solvers can be highly optimized by analyzing and exploiting the topology of this graph. Roughly, the general solution of *n *linear (nonlinear) equations is at most (at least) an effort in the order O(*n*^3^) which becomes prohibitive when *n *grows beyond a certain limit. If an equation system can be decomposed into *k *consecutive subsystems of dimension *n*_1 _+ ... + *n*_*k *_= *n *the effort consequently reduces to $O(n_{1}^{3})+...+O(n_{k}^{3})$ which entails a tremendous speedup if *n*_1_,..., *n*_*k *_≪ *n*.

Fig. 1 illustrates this idea on the small example network. Obviously, the network nodes *x*_1_, *x*_2 _and *x*_3 _are cyclically linked which means that the corresponding balance equations are closely coupled and have to be solved simultaneously, e.g. by Gaussian elimination, or – in the nonlinear case – the application of the Newton algorithm [47]. The same holds for nodes *x*_4_, *x*_5 _and *x*_6_. Last, the node *x*_7 _is linked to both cycles by a *hyper-edge*.

Obviously, it is not necessary to solve all seven equations in a single O(7^3^) run because *x*_1_, *x*_2 _and *x*_3 _do not depend on x_*j*_, *j *= 4,...,7 which are lying *downstream *in the network. Consequently, *x*_1_, *x*_2_, and *x*_3 _are to be computed in O(3^3^) steps first, followed by another O(3^3^) computation for *x*_4_, *x*_5_, and *x*_6_. Finally, *x*_7 _can be determined from the predecessor node solutions for *x*_2 _and *x*_6 _by evaluating the quadratic term *x*_7 _= (*v*_5_·*x*_2_·*x*_6_)/*v*_6 _= *x*_2_·*x*_6 _in O(1) steps. For the simple example this is already six times faster than the unpartitioned solution, but for realistic networks the speedup factor is usually much higher (factor 10^2 ^– 10^5^; depending on network connectivity).

#### Graph isomorphisms and tracing of labeled compounds

A closer inspection shows that, due to their very special origin, ILNs are not just ordinary computational graphs. Because of the combinatorial way an ILN is constructed from a much smaller atom network the ILN is expected to contain redundancy to a great extent. In this context cyclic sub-networks, i.e. the sets of cyclically interdependent nodes, are of special interest. For the small network shown in Fig. 1 such node sets are {*x*_1_, *x*_2_, *x*_3_} and {*x*_4_, *x*_5_, *x*_6_}. As a major result it will turn out that ILNs naturally break up into a high number of these sets, always yielding an enormous computational speedup when solving the associated LBEs.

Moreover, many of the cyclic components are isomorphic. In the example network in Fig. 1 the two cycles describe isomorphic components since the nodes matched by *x*_1 _≡ *x*_4_, *x*_2 _≡ *x*_5_, and *x*_3 _≡ *x*_6 _are connected by the same flux values. Since these isomorphic components essentially describe identical subsystems of the equations, their identification gives precise statements about the redundancy contained in the ILN. By preventing repeated solution of these isomorphic subsystems additional speedup is possible.

Last but not least, the global topological analysis of ILNs yields a complete understanding of the flow of labeled material in the network which is extremely helpful information for experimental design. Hitherto, this information could only be obtained for specific networks by the manual tracing of labeled compounds through the network.

The focus of this contribution is on topological network analysis and not on the algorithmic details of equation formulation and solution which will be treated in a future publication. Nevertheless, a first prototype of a topology-based LBE solver has been implemented which demonstrates that a speedup factor of about 1000 can be achieved for a realistic *E. coli *carbon ILN. This speedup will already solve most of the current performance problems mentioned in the introduction. Moreover, to keep the exposition short, the ILNs are described from the viewpoint of the cumomer method [14], although the introduced concepts apply to any of the above mentioned types of network graphs.

## Methods

### Networks in MFA

The formal structure of ILNs is well documented in literature [14,39,42,43,48]. However, to give a precise definition of the terminology and nomenclature used in this contribution a brief summary of the basic concepts is given in this section.

#### Metabolic networks and isotope labeling networks

The subject of isotope-based MFA and the context of this work is the quantification of the material flow between a cell's intra-cellular metabolite pools. MFA is based on the forward simulation of an ILN, which determines how metabolic reactions distribute an isotope labeling, taken from the substrate (e.g. a ^13^C labeling taken from a pool of glucose molecules), among the cell's metabolic pools when the reaction rates (flux values) are assumed to be known.

**Figure 2:**
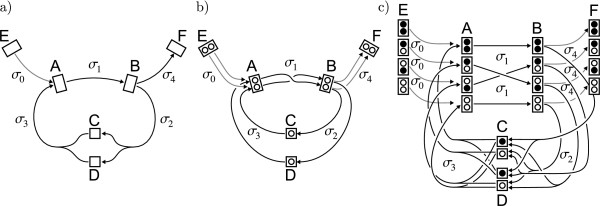
**Example metabolic network**. a) reaction network, b) atom transition network, c) isotopomer network. The number of compartments and transitions in the isotopomer network increases exponentially with the length of atom backbones.

The metabolite network (cf. Fig. 2a) is the most basic graph representation used in this context and shows how metabolic pools are connected by the biochemical reactions. These networks can be formalized by hyper-graphs since they contain hyper-edges for bimolecular reactions. The representation in Fig. 2a lacks the information about how atoms are transported between the pools, which is crucial for MFA. This missing information about the reactions atom transitions (i.e. their *permutation property*) is contained in the atom transition network (cf. Fig. 2b). As opposed to the metabolic network the atom transition network can be represented by a standard directed graph.

Unfortunately, labeling data are seldomly directly related to single atom positions. To explain NMR and MS measurements, a more detailed representation of molecular labeling states is necessary. For this purpose, a third graph representation is introduced – the *isotopomer network*.

#### Isotopomer networks

*Isotopomers *are isotopic isomers, i.e. molecular entities that differ solely in their isotopic composition. Unlike the atom transition networks, isotopomer networks are a well-suited basis for the evaluation of ILEs because they allow the balancing of metabolic pools in all possible labeling states (cf. Fig. 2c). From a structural viewpoint, isotopomer networks do not contain more information than the carbon atom networks because the one is derived from the other.

The simulation of LBEs in terms of isotopomers suffers from a combinatorial explosion of the number of nodes, because a metabolite with *s *atom positions available for isotopic labeling is expanded into a set of 2^*s *^different isotopomers. Even for a small atom transition network this may result in a large ILN graph; depending on the size of the molecules included.

Fortunately, the whole ILN can be transformed into a network of *Cumomers *(cumulative isotopomers), henceforth called cumomer labeling network (CLN) which enables the partitioning of the network graph. Since the resulting partitions contain only unimolecular transitions and special degenerated types of bimolecular transitions, the corresponding system of cumomer balance equations is linear in its unknowns [42].

#### Cumomer networks

A cumomer is a virtual particle which is called *cumulative *because it describes a set of isotopomers. While each atom position in an isotopomer is either in state *labeled*/● or *unlabeled*/○ the atom positions in cumulative isotopomers are in one of the two states *labeled*/● or *don't care*/. Here, the special state *don't care*/ represents a wildcard which is expanded into the set of states {•, ○} so that cumomers containing these wildcards describe sets of isotopomers (e.g. ). Likewise, the cumomer transitions described by metabolic reactions can be understood as one-to-one mappings between sets of isotopomers.

In other words, the labeled atom positions in a cumomer describe a labeled fragment which is shared among its set of isotopomers. From this perspective the reactions in a cumomer network describe how labeled fragments of certain size are transported by the metabolic reactions. These fragments may be permuted by the metabolic reactions so that they are not necessarily contiguous (e.g. ). Paths through the cumomer network graphs can be characterized by the property that a certain subset of atoms is retained by a chain of metabolic reactions (cf. Fig. 6).

**Definition 1 **(cumomer weight). *If the term weight is used in connection with cumomers it shall denote the fragment size which coincides with the number of atom positions that are in state labeled*/●.

The weight of cumomers induces the partitioning of the cumomer space:

• In weight-0-cumomers all atom positions are occupied by wildcards () and a cumomer of weight 0 and size *s *always describes the full set of 2^*s *^isotopomers in all possible labeling states, i.e. the metabolic network itself.

• Another important special case is the network described by the transitions between weight-1-cumomers. This network describes the transport of single-atom fragments, and therefore the atom transition network. Since all balanced atoms in the metabolite pools originate in the network's input pools the transitions in this special network connect every atom in the system with one or more atoms of the substrate pools.

• Weight-*k*-networks constitute the subset of reactions that transport a labeled fragment of size *k*. A metabolite with *k *atom positions has exactly one representative on level *k *and none on the weight levels > *k*.

#### Assembling cumomer networks

The change from isotopomer to cumulative isotopomer pools raises the important question how the cumulation of isotopomer pools affects the transitions in the network and therefore the topology of network graphs. As shown by the following rules the translation of unimolecular transitions, as well as bimolecular assembly transitions, is straight forward. For bimolecular split transitions the central concept of *weight conservation *applies which prevents transitions that would reduce weight, i.e. distribute the labeling of an educt on more than one product. The rules for the translation of isotopomer networks into cumomer networks can be summarized as follows (see [42] for an alternate formulation):

**Definition 2 **(basic translation rules). *The transformation of isotopomer transitions into cumomer transitions can be performed by application of the following basic translation rules*:

*1. Replace all unlabeled atom positions *○ *by wildcard labels *.

*2. If transitions associated with splitting reactions violate the weight conservation constraint, i.e. more than one product has a non-zero weight, replace the transition by an efflux into a virtual sink pool *Ω.

*3. Remove all weight-0-cumomers together with all incident edges from the network*.

**Figure 3:**
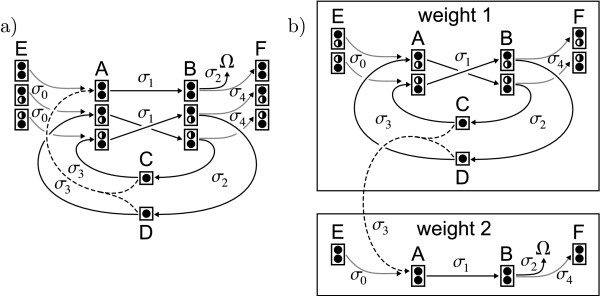
**Cumomer networks**. a) Cumomer network for the carbon atom transition network in Fig. 1. b) natural cascaded structure of cumomer networks given by the weight of cumomers.

Application of these rules on the example network in Fig. 2 leads to the cumulative isotopomer network in Fig. 3a: rule no. 1 replaces unlabeled positions by wildcard labelings. Rule no. 2 replaces the splitting reaction  → C• + D• by an efflux into the special pool Ω. Finally the weight-0-cumomers  and the edges leading to and coming from these pools are removed from the network by rule no. 3.

Rule no. 3 can be justified by intuition: in a weight-0-cumomer every position is occupied by a wildcard . In other words: the whole cumomer is in state *don't care*. Since a weight-0-cumomer describes a fragment of size zero and is the complete set of isotopomers comprising all possible labeling states they do not contain any information at all and can be removed from the network (again, see [42] for details).

### Structure of this contribution

So far, it has been shown how isotopomer networks are converted into cumomer networks. In contrast to the ILNs, the CLNs presented in the previous section possess a *weight conservation *property which establishes a subdivision of cumomer networks and a partitioned solution. The following section will briefly review this long-known property in order to pave the way for two new graph-theoretical features, proven in the subsequent parts.

The first new feature unveiled is *decreasing connectivity with increasing cumomer-weight*, which describes an incremental break-up of cumomer networks for larger fragments. The second new feature is the inherent *isomorphism of subgraphs*, which can be characterized as an effect introduced by the combinatorial fashion the isotopomer and cumomer networks are built. Isomorphism also affects the cumomer network graph's strongly connected components which is further exposed at the end of this section.

Section *Example network *then shows the practical consequences of the obtained results for a realistically sized *E. coli *^13^C labeling network, consisting of a cascaded cumomer network containing about 10^4 ^cumomer nodes. In sec. *Global connectivity and reachability *an important application of the connectivity concepts is given by the global study of connectivity and reachability of network nodes. The general concept of a *reduced network *is introduced which explains the formation of a certain labeling state in a desired subset of metabolic pools. It turns out that the dimension of the *E. coli *cumomer network can be strongly reduced depending on the measurement configuration and the present input labeling mixture.

## Results and discussion

### Topological properties of cumomer networks

This section summarizes the topological consequences of the transformation rules presented in sec. *Cumomer networks *and introduces new theoretical results. For the comprehension of the following sections basic knowledge of graph theory is assumed and only the basic concepts shall be briefly introduced here (see e.g. [49] for an introduction).

In order to describe the topological properties of the cumomer cascade it is helpful to introduce the notion of a *labeled digraph*:

**Definition 3 **(labeled digraph). *A *labeled directed graph (labeled digraph) *G *= (*V*, *E*, Σ_*E*_) *is a structure which consists of a set V of "nodes" labeled *1, 2,..., *n, a set *Σ_*E *_*of "edge labels" and a mapping E *⊆ *V *× Σ_*E *_↦ *V called "edges"*.

In contrast to the commonly used definitions for directed graphs this definition allows to have multiple edges with different edge labels between a pair of nodes.

#### The cumomer cascade

The first important consequence of the rules presented in Def. 2 is that cumomer networks have a *natural partitioning *by weight [42]. By the application of rule no. 2 all weight-reducing transitions induced by splitting reactions are removed from the network. Since all remaining transitions are either unimolecular, weight conserving bimolecular splitting reactions or bimolecular assembly reactions, all transitions either conserve or increase the weight of cumomers.

Grouping cumomers by weight leads to a natural partitioning into cascaded network graphs (^1^*G*, ^2^*G*,...,^*s*^*G*) with ^*k*^*G *= (^*k*^*V*, ^*k*^*E*, Σ_*E*_) for 1 ≤ *k *≤ *s *(cf. Fig. 3b). Within these graphs ^*k*^*G*, transitions ^*k*^*E *are weight-conserving [42]:

• **Unimolecular transitions **are always weight-conserving. For example, in Fig. 3, the unimolecular transitions induced by reactions *σ*_0 _ and *σ*_1 _ transport the complete isotope labeling from the educt to the product cumomer.

• **Splitting transitions **transport the full weight of the educt cumomer to a single product cumomer. In the splitting reactions *σ*_2 _of Fig. 3, this holds for the transitions  → C• and  → D•. There is no such transition for  because distributing the weight on C• and D• is not allowed.

• In **assembly transitions**, the full weight of the product cumomer is originated in only one educt cumomer. For reaction *σ*_3 _in Fig. 3 this holds for transitions C• →  and D• → . The transition C•, D• →  creates a product of higher weight and connects the cascade levels.

If, for an assembly transition, there is more than one educt cumomer with weight > 0, the assembly reaction induces a *hyper-edge *which connects multiple educt cumomers with one product cumomer by crossing levels of the cascade. Within the levels of the cascade, the remaining assembly transitions are weight-conserving.

From the viewpoint of cumomer balance equations hyper-edges are treated as ordinary influxes from source pools and do not require special treatment. Clearly, these hyper-edges are not covered by Def. 3, since edges are defined to connect exactly two nodes. Because the weight level cumomer network graphs ^*k*^*G *discussed in the following are free of hyper-edges, the associated hyper-graphs are not needed, and hence not formally introduced here.

#### Decreasing connectivity with increase of weight

The following lemma states that weight level 1 is always the most connected graph in the cumomer cascade and the global connectivity decreases with increasing cascade level:

**Lemma 1 **(decreasing connectivity). *Let *^*k*^*G *= (^*k*^*V*, ^*k*^*E*, Σ_*E*_) *denote the cumomer cascade's graph on weight level k. Let *ε(kG)def¯¯|Ek|/|Vk|*denote the average node degree (i.e. the average number of edges leaving a node), which is a measure for the graph's connectivity. With increasing weight level k the average node degree decreases monotonically, i.e. ε*(^*k*+1^*G*) ≤ *ε*(^*k*^*G*) *for k *= 1, 2,..., *s *- 1.

The proof can be found in the appendix. Tab. 1 illustrates the consequences of lemma 1 on three minimal networks, each consisting of a single reaction. The '#'-notation used for the cumomers in this example was introduced in [42] and is employed throughout the whole contribution: the "1" denotes a labeled/● atom position and the "x" a don't-care/ atom position, while the order of labeling positions •/ characterizes a reaction's specific atom transport properties. Tab. 1 shows the cumomer nodes and transitions on the individual weight levels for each of the minimal networks. For the unimolecular reaction, connectivity does not change over the weight levels while for the bimolecular types connectivity decreases monotonically.

**Table 1:** Consequences of lemma 1

weight	A#abc → B#acb	*ε*(^*k*^*G*)
1	A#1xx → B#1xx	$\frac{1}{2}$
	A#x1x → B#xx1	
	A#xx1 → B#x1x	

2	A#11x → B#1x1	$\frac{1}{2}$
	A#1x1 → B#11x	
	A#x11 → B#x11	

3	A#111 → B#111	$\frac{1}{2}$

weight	B#abc → C#a + D# cb	*ε*(^*k*^*G*)

1	B#1xx → C#1	$\frac{1}{2}$
	B#x1x → D#x1	
	B#xx1 → D#1x	

2	B#11x	$\frac{1}{4}$
	B#1x1	
	B#x11 → D#11	

3	B#111	0

weight	C#a + D#bc→ E#bac	*ε*(^*k*^*G*)

1	C#1 → E#x1x	$\frac{1}{2}$
	D#1x → E#1xx	
	D#x1 → E#xx1	

2	E#11x	$\frac{1}{4}$
	E#x11	
	D#11 → E#1x1	

3	E#111	0

Three different networks consisting of a single reaction. Connectivity *ε*(^*k*^*G*) decreases monotonically with increasing weight level *k*.

A reaction's permutation property not only defines which pairs of cumomer nodes are connected by an edge, it also defines how many edges can be found on the individual network levels. To quantify this observation, it is helpful to introduce the term *width of a reaction*:

**Definition 4 **(width of a reaction, width of a reaction sequence). *The term *width of a reaction *shall denote the maximum size of a fragment transported between the reactants on both sides of the reaction*:

*1*. The width *of an unimolecular reaction *R : A#*a*_1_*a*_2_... *a*_*s *_⇌ B#*b*_1_*b*_2_... *b*_*s *_*simply corresponds to the number s of transported labeling positions, i.e*. $width{\left(R,\text{A},\text{B}\right)}^{\underline{\underline{def}}}s$.

*2. For any bimolecular reaction *R : A#*a*_1_*a*_2_... *a*_*q *_+ B#*b*_1_*b*_2_... *b*_*r *_⇌ C#*c*_1_*c*_2_... *c*_*s *_*where q *+ *r *= *s the width is defined by the number of labeling positions transported between the pools, i.e*. $width{\left(R,\text{A},\text{C}\right)}^{\underline{\underline{def}}}\min\{q,s\}=q\;and\;width{\left(R,\text{B},\text{C}\right)}^{\underline{\underline{def}}}\min\{r,s\}=r$.

*3. The *width of a sequence of reactions, $R_{1},...,R_{\ell }$, *which transport molecular fragments through pools *P_0_,..., P_ℓ _*are inductively defined as the maximal size of a transported fragment, i.e. the minimum of widths of the involved reactions which is given by the size of the smallest atom backbone in the reaction sequence: *$width(R_{1},...,R_{\ell },{\text{P}}_{0},...,{\text{P}}_{\ell }){)}^{\underline{\underline{def}}}\min_{i=1,...,\ell }\{width(R_{i},{\text{P}}_{i-1},{\text{P}}_{i})\}$

*4. width*(R, A, B) = *width*(R, B, A), *i.e. the width of a reaction shall not depend on its reversibility*.

**Figure 4:**
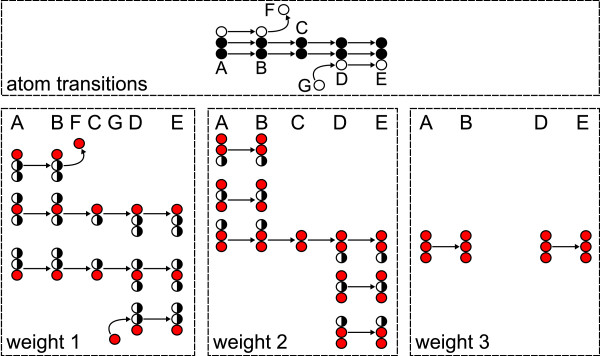
**Illustration of lemma 3**. Lemma 3 illustrated: multiple isomorphic copies of the original path A → E (top) on different weight levels. Assembly transitions connecting the weight levels and effluxes into Ω are omitted.

Example: in Fig. 4 (top) the width of the unimolecular reactions A → B and D → E is three since three atom positions are transported. The width of the bimolecular splitting reaction B → F + C is one for B → F and two for B → C. The width of the reaction sequence A → ... → E is the minimum of the individual widths, i.e. the reaction sequence transports a fragment of size two.

**Lemma 2 **(number of edges induced by reactions). *The number of edges induced by a metabolic reaction on a specific weight level k of the cumomer cascade depends on its width*:

*1. An unimolecular reaction *R : A#*a*_1_*a*_2 _... *a*_*s *_→ B#*b*_1_*b*_2 _... *b*_*s *_*induces*

$\left(\begin{array}{c} width(R,\text{A},\text{B})\\ k \end{array}\right)=\left(\begin{array}{c} s\\ k \end{array}\right)$*edges on weight level k of the cascade*.

*2. A bimolecular reaction *R : A#*a*_1_*a*_2 _... *a*_*q *_+ B#*b*_1_*b*_2 _... *b*_*r *_⇌ C#*c*_1_*c*_2 _... *c*_*s *_*induces*

$\left(\begin{array}{c} width(R,\text{A},\text{C})\\ k \end{array}\right)+\left(\begin{array}{c} width(R,\text{B},\text{C})\\ k \end{array}\right)=\left(\begin{array}{c} q\\ k \end{array}\right)+\left(\begin{array}{c} r\\ k \end{array}\right)$*edges on weight level k of the cascade*.

The simple proof is left to the reader. Again, Tab. 1 can be used to illustrate lemma 2: the unimolecular reaction induces $\left(\begin{array}{c} 3\\ k \end{array}\right)$ edges and both bimolecular reactions induce $\left(\begin{array}{c} 1\\ k \end{array}\right)+\left(\begin{array}{c} 2\\ k \end{array}\right)$ edges ("→") for *k *= 1, 2, 3. Thus, lemma 2 shows that the number of edges induced by a metabolic reaction on a certain level of the cumomer cascade depends only on the number of atoms transported between the individual educt-product pairs.

#### Isomorphic subgraphs within the cascade

Although the cumomers originating in the same metabolic pool can be found on different weight levels of the cascade, they are connected by edges with the same flux labels Σ_*E *_that connect the underlying metabolic pools. This similarity of graphs can be formalized by the notion of graph isomorphism:

**Definition 5 **(graph isomorphism). *A subgraph G*_*s*_= (*V*_*s*_, *E*_*s*_, Σ_*E*_) *of a graph G *= (*V*, *E*, Σ_*E*_) *is given by a set of nodes V*_*s *_⊂ *V and an associated set of edges E*_*s *_⊂ *E where *(*u*, *σ*, *v*) ∈ *E*_*s *_⇔ (*u*, σ, *v*) ∈ *E *∧ *u*, *v *∈ *V*_*s*_. *Two subgraphs G*_1 _= (*V*_1_, *E*_1_, Σ_*E*_) *and G*_2 _= (*V*_2_, *E*_2_, Σ_*E*_) *are isomorphic if and only if there exists a bijective function (permutation) π *: *V*_1 _↦ *V*_2 _*which relabels the nodes of V*_1 _*with the properties V*_2 _= *π*(*V*_1_), *and *(*p*, *σ*, *q*) ∈ *E*_1 _⇔ (*π*(*p*), *σ*, *π*(*q*)) ∈ *E*_2_. *The isomorphism between G*_1 _*and G*_2 _*shall be informally denoted as G*_2 _= *π*(*G*_1_) *resp. G*_1 _= *π*^-1^(*G*_2_).

*A directed path *of length ℓ from a node *p *to a node *r *is a sequence of edges (*e*_1_, *e*_2_,..., *e*_ℓ_) such that *e*_*i *_= (*q*_*i*-1_, *σ*_*i*_, *q*_*i*_), *q*_0 _= *p*, and *q*_ℓ _= *r*. The following lemma, whose proof can be found in the appendix, quantifies the appearance of isomorphic subgraphs defined by a directed path's nodes *q*_0_,..., *q*_ℓ_:

**Lemma 3 **(isomorphic paths). *Given a sequence of reactions *$R_{1},...,R_{\ell }$*that transports a (not necessarily continuous) fragment through the pools *P_0_,..., P_ℓ_. *Assume that all participating metabolite backbones in this path can be labeled on at least s atom positions, i.e. width*($R_{1},...,R_{\ell }$, P_0_,..., P_ℓ_) = *s*.

*Then the original reaction sequence induces *$\left(\begin{array}{c} s\\ k \end{array}\right)$*disjoint isomorphic paths on levels k *= 1,..., *s of the cascade (i.e*. 2^*s *^- 1 *disjoint isomorphic paths in total) which are "copies" of the original reaction sequence and do not share common cumomer nodes (i.e. disjoint isomorphic paths)*.

Fig. 4 illustrates lemma 3 on a sequence of reactions transporting a fragment of size two from pool A to pool E: A#abc → B#abc, B#abc → F#a + C#bc, C#ab + G#c → D#abc, D#abc → E#abc: Pool C has the shortest atom backbone within the reaction chain and limits the fragment size to two atoms. As lemma 3 predicts, there are two isomorphic copies of the reaction chain on level one of the cascade and a single copy on level two. There are no isomorphic paths left on weight level three because of the disappearance of pool C. Summing up, there are 2 + 1 + 0 = 2^2 ^- 1 isomorphic paths from pool A to E in the cascade. For the shorter sub-path from pool A to B, which carries a fragment of three atoms, there are 3 + 3 + 1 = 2^3 ^- 1 isomorphic paths in the cascade.

Isomorphic paths naturally extend to isomorphic subgraphs. Likewise a reaction sequence that transports a fragment of size *s *leads to $\left(\begin{array}{c} s\\ k \end{array}\right)$ isomorphic paths on weight level *k *of the cascade, there are $\left(\begin{array}{c} s\\ k \end{array}\right)$ isomorphic copies of a metabolic network's subgraph iff all reactions transport a fragment of at least size *s*. It follows, that a connected subgraph of a metabolic network induces connected isomorphic subgraphs in the cumomer cascade as long as weight level *k *does not exceed the size *s *of the shortest fragment transported between the metabolic pools.

#### Components of cumomer network graphs

As motivated for the illustrative example at the beginning, the smallest structural units of an ILN are the subgraphs containing cycles. In order to describe how labeled fragments cycle through the network this informal conception can be formalized by introducing the notion of strongly connected components (SCCs):

**Definition 6 **(strongly connected component). *A subgraph G*_*s *_= (*V*_*s*_, *E*_*s*_, Σ_*E*_) *of labeled digraph G *= (*V*, *E*, Σ_*E*_) *is strongly connected if there exists a directed path *(*e*_1_,..., *e*_ℓ_) ∈ $E_{s}^{*}$*between any pair of its nodes *(*p*, *q*) ∈ *V*_*s*_. *G*_*s *_*is a strongly connected component of G if it cannot be enlarged by adding more nodes and associated edges without losing the property of being strongly connected*.

Again, Fig. 1 illustrates the concept: obviously, the nodes within the two cycles are strongly connected. Starting at a node of the right cycle there is no way to reach the nodes of the left cycle. Consequently, there are two groups of strongly connected nodes. Since adding *x*_0 _or *x*_7 _to either groups would break their strongly connectedness the two cycles already represent the SCCs of the network. By convention, a solitary node shall be treated as a SCC consisting of a single node, hence the example network graph consists of four SCCs.

The *component graph *is an important graph associated with a graph's decomposition into SCCs. Its nodes are the SCCs of the original graph and it contains a directed edge between two nodes if there is at least one edge in the original graph which connects nodes of the corresponding SCCs. Hence, the component graph is acyclic and there exists an order on its nodes (i.e. the original graph's SCCs), which is called *topological sort*. The component graph is topologically sorted iff for all edges from *i *to *j *node *i *appears before node *j *[49]. A topological sort for the example network in Fig. 1 is (*x*_0_, {*x*_1_, *x*_2_, *x*_3_}, {*x*_4_, *x*_5_, *x*_6_}, *x*_7_). The following definition introduces the notion of a *connected component *(CC). The CCs of a digraph describe a weaker form of connectivity:

**Definition 7 **(connected component). *A connected component is a subgraph G*_*s *_= (*V*_*s*_, *E*_*s*_, Σ_*E*_) *of a labeled digraph G *= (*V*, *E*, Σ_*E*_) *which were a SCC if its edges E*_*s *_*could be traversed in any direction*.

Obviously, a digraph is said to be connected if it consists of a single CC. In particular CCs describe a partition of a digraph's nodes and edges into disjoint subsets, i.e. disconnected subgraphs. Any of these CCs is either a SCC or can be further split into a set of SCCs.

### *E. coli *example network

**Figure 5:**
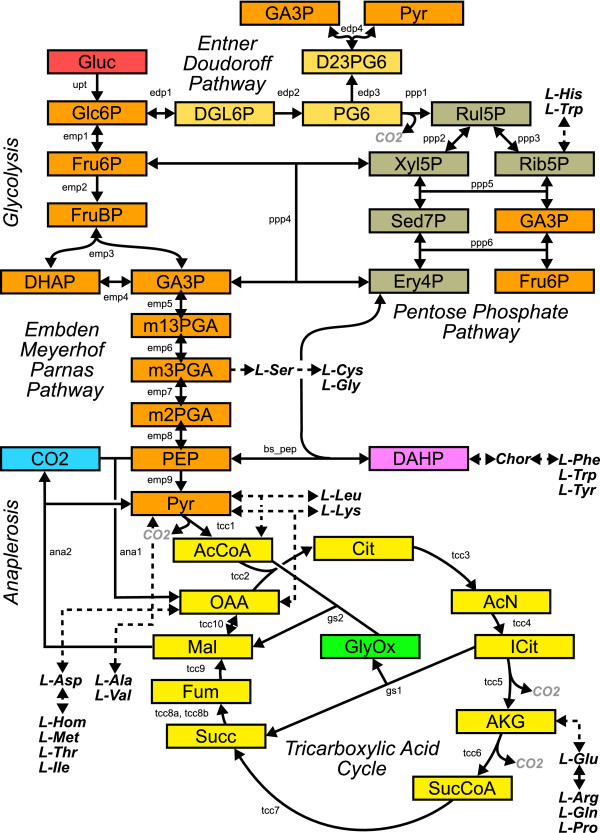
***E. coli *example network**. The central metabolism of *E. coli *with attached biosynthesis pathways.

This section illustrates the practical consequences of the theoretical results of the previous sections on the basis of a realistic ^13^C labeling network which models the central metabolism and the attached biosynthesis pathways of *E. coli *(cf. Fig. 5, Additional file 1).

Two variants of this metabolic network will be discussed in the following. In the first variant, network "A", the reversibilities of metabolic reactions are assumed to be realistic, i.e. the majority of metabolic reactions is considered to be bidirectional. Tab. 2 summarizes the setup of network "A". The detailed reaction network can be found in the supplementary material. In the course of this section network variant "B" is introduced. This extreme variant of network "A" shares the same metabolic reactions, however all reactions are considered to be unidirectional. Although this assumption is certainly not realistic, the comparison of both network variants is valuable for emphasizing the topological effects of reversible reactions.

**Table 2:** Configuration of the example network "A"

pools	87 (3 sources, 30 sinks, 54 inner)
reactions	94 (3 input, 30 output, 61 inner reactions)
inner reactions	61 (19 unidirectional, 42 bidirectional)
size of largest carbon backbone	11 (Trp)
reactions considered as unidirectional	emp2 emp9 edp2 edp3 ppp1 tcc1 tcc2 tcc3 tcc4
	tcc5 tcc6 tcc7 tcc8a tcc8b tcc9 ana1 ana2 gs1 gs2

SCCs and CCs represent recognizable subsystems that can be found throughout the weight levels of the whole cumomer cascade. For this reason (S)CCs are an ideal test case to illustrate the theoretical results of the previous section.

#### Incremental break-up of the network graphs

The number of weight levels of the corresponding cumomer cascade is given by the size of the largest carbon atom backbone in the system – the amino acid L-tryptophan (pool Trp). The cascade's subgraphs ^*k*^*G *= (^*k*^*V*, ^*k*^*E*, Σ_*E*_) are characterized in Tab. 3: column |^*k*^*V*| contains the number of nodes for the respective level of the cascade and therefore the number of cumomers of weight *k*. As predicted by lemma 1, the graphs' connectivity *ε*(^*k*^*G*) decreases monotonously with increasing weight level *k*.

**Table 3:** Topological analysis of the cumomer cascade of example network "A" (realistic flux reversibilities)

level *k*	|^*k*^*V*|	|^*k*^*E*|	CCs (avg. size)		*ε*(^*k*^*G*)	distribution of SCC sizes
1	263	615	1	263.00	2.34	12: 1:7x 3:1x 4:2x 70:1x 175:1x
2	633	1089	133	4.76	1.72	231: 1:175x 2:11x 3:7x 4:16x 5:4x 6:6x 7:4x 8:1x 10:1x 12:1x 18:1x 23:1x 50:1x 64:1x 82:1x
3	1003	1391	397	2.53	1.39	531: 1:424x 2:21x 3:15x 4:25x 5:4x 6:24x 7:8x 8:1x 10:5x 18:1x 23:2x 50:1x
4	1201	1414	658	1.83	1.18	747: 1:625x 2:22x 3:23x 4:19x 5:3x 6:44x 7:5x 10:5x 23:1x
5	1158	1150	770	1.50	0.99	802: 1:703x 2:15x 3:24x 4:7x 5:2x 6:48x 7:1x 10:2x
6	896	699	680	1.32	0.78	685: 1:626x 2:6x 3:19x 4:1x 5:1x 6:32x
7	532	290	447	1.19	0.55	447: 1:422x 2:1x 3:12x 6:12x
8	229	72	209	1.10	0.31	209: 1:202x 3:5x 6:2x
9	67	8	65	1.03	0.12	65: 1:64x 3:1x
10	12	0	12	1.00	0.00	12: 1:12x
11	1	0	1	1.00	0.00	1: 1:1x

Likewise the global connectivity *ε*(^*k*^*G*) decreases with increasing cascade level *k *(cf. lemma 1) the SCCs have a tendency to break up into smaller SCCs: weight level one consists of a single CC which contains large SCCs. With increasing weight level the number of CCs increases, while the average number of nodes per CC decreases. The higher levels of the cascade are mainly disconnected, e.g. level 8 has 229 nodes but only 9 smaller SCCs and 202 disconnected nodes. Level 9 is completely disconnected except a single SCC of size 3. In level 10 finally only detached nodes remain.

#### The impact of bidirectional reactions

The presence and size of SCCs is mainly governed by the reversibility of metabolic reactions: every bidirectional unimolecular reaction trivially causes two nodes to be strongly connected and every linear chain of bidirectional reactions results in a strongly connected subgraph. Only few SCCs in the cascade's graphs are actually caused by cyclic reaction chains in metabolic pathways. To quantify this statement an extreme variant of the *E. coli *network, referred to as network "B" in the following, is examined where all metabolic reactions are assumed to be unidirectional.

**Figure 6:**
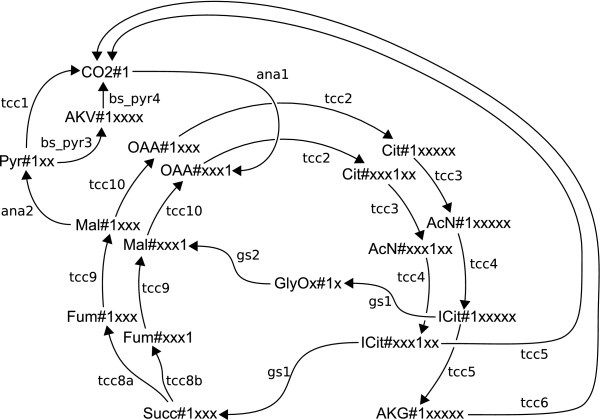
**SCC of network "B" (TCA cycle)**. A SCC of network "B" found in the TCA cycle on network level one. The pool SucCoA (Succinyl coenzyme A) is bypassed by GlyOx (Glyoxylate) and CO2 (CO_2_).

The results are shown in Tab. 4. At first, it is important to note that this modified network has the same CCs, i.e. the restriction of reversibilities does not disconnect the network graphs. Furthermore, the carbon atom network (weight level one) is still connected in the sense that every carbon atom in the system can be reached by a path coming from the substrate atoms. For this reason, the SCCs of network "B" describe (nontrivial) cyclic reaction chains, Fig. 6.

**Table 4:** Topological analysis of the cumomer cascade of example network "B" (unidirectional fluxes)

level *k*	|^*k*^*V*|	|^*k*^*E*|	CCs (avg. size)		*ε*(^*k*^*G*)	distribution of SCC sizes
1	263	354	1	263.00	1.35	217: 1:212x 3:3x 18:1x 24:1x
2	633	620	133	4.76	0.98	614: 1:610x 3:3x 14:1x
3	1003	771	397	2.53	0.77	1001: 1:1000x 3:1x
4	1201	753	658	1.83	0.63	1201: 1:1201x
5	1158	591	770	1.50	0.51	1158: 1:1158x
6	896	352	680	1.32	0.39	896: 1:896x
7	532	145	447	1.19	0.27	532: 1:532x
8	229	36	209	1.10	0.16	229: 1:229x
9	67	4	65	1.03	0.06	67: 1:67x
10	12	0	12	1.00	0.00	12: 1:12x
11	1	0	1	1.00	0.00	1: 1:1x

The large SCC on level one of dimension 24 covers the full TCA cycle and describes two independent paths of single carbon atoms through the cycle's metabolites. These two paths are connected by the GlyOx#x1 cumomer and the anaplerotic reactions. A similar cycle is described by the SCC of size 18 shown in Fig. 6 where pool SucCoA is bypassed completely by a path through cumomer GlyOx#1x. This bypass via the GlyOx pool has the ability to transport a fragment of size two and is found as the SCC of size 14 on level two of the cascade (cf. Fig. 6). Since there is no SCC on weight level two which includes cumomers of pool SucCoA, only a single carbon atom may be kept on a cyclic path through all the pools of the TCA cycle.

The graphs on weight levels 10 and 11 contain no edges at all, which indicates that the cumomers of the corresponding weight are fed from the lower levels of the cascade as the product of assembly reactions.

#### Isomorphic SCCs

As stated above, the SCCs of the cumomer network describe cyclic pathways of certain labeled fragments. On the other hand SCCs represent distinguished subsystems of cumomer network graphs they are a convenient test case to demonstrate the consequences of lemma 3, i.e. a cumomer network can be expected to contain isomorphic SCCs if only a strongly connected subgraph transports a fragment of more than one atoms.

The standard approach to find a graph isomorphism, i.e. to compute at least one possible node renaming *π*, is to perform a clever backtracking through all |*V*|! possible node assignments [50]. Although, there is no polynomial-time algorithm known for the general graph isomorphism problem this standard algorithm performs well, especially if graphs are as loosely connected as the cumomer network graphs.

The key to even more efficient isomorphism testing is to partition the node set into equivalence classes such that two nodes in different classes cannot possibly be mistaken for each other [51]. For cumomer network graphs, the basic equivalence classes are a-priori given by the nodes' origin in the underlying metabolic pools. These classes can be further split into subclasses by comparing node degree and labels of incident edges. Therefore, the detection of isomorphic SCCs in cumomer network graphs is efficient in practice.

**Figure 7:**
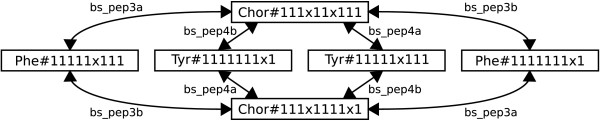
**Example SCC with isomorphic copies**. A SCC found in network "A" that transports a fragment of size eight. Isomorphic copies of this SCC can be found on weight levels 2 through 8 (cf. Tab. 3).

Because of the greater practical relevance, and the more interesting SCC distributions the weight level graphs of network "A" were analyzed for graph isomorphisms. Tab. 5 shows the results of this analysis. The surprising outcome is that there are only very few unique SCCs: the largest graph, ^4^*G*, contains 122 SCCs with ≥ 2 nodes but only 12 distinct SCC equivalence classes. The largest equivalence classes found in graphs ^2^*G *through ^8^*G *belong to a single isomorphic SCC of size six that describes transitions between cumomers of the pools Chor, Phe and Tyr which transports a fragment of eight carbon atoms, shown in Fig. 7. This example is exceptional, since SCCs tend to break up with increasing cascade level – in fact, in ^1^*G *this subsystem is embedded in a larger SCC. Therefore, the SCC distribution cannot be expected to follow lemma 3 precisely.

**Table 5:** Equivalence classes in the SCC distributions

level k	s'up	s'up (iso)	SCC equivalence classes; notation: [*SCC size*]_*class size*_
1	3x	3x	4: [3]_1_, [4]_2_, [70]_1_, [175]_1_
2	263x	264x	20: [2]_3_, [2]_4_, [2]_4_, [3]_1_, [3]_6_, [4]_6_, [4]_10_, [5]_2_, [5]_2_, [6]_6_, [7]_1_, [7]_3_, [8]_1_, [10]_1_, [12]_1_, [18]_1_, [23]_1_, [50]_1_, [64]_1_, [82]_1_
3	5877x	6899x	16: [2]_3_, [2]_4_, [2]_14_, [3]_15_, [4]_10_, [4]_15_, [5]_1_, [5]_3_, [6]_24_, [7]_2_, [7]_6_, [8]_1_, [10]_5_, [18]_1_, [23]_2_, [50]_1_
4	55171x	115504x	12: [2]_1_, [2]_1_, [2]_20_, [3]_23_, [4]_5_, [4]_14_, [5]_3_, [6]_44_, [7]_1_, [7]_4_, [10]_5_, [23]_1_
5	104357x	608955x	8: [2]_15_, [3]_24_, [4]_1_, [4]_6_, [5]_2_, [6]_48_, [7]_1_, [10]_2_
6	86791x	674787x	5: [2]_6_, [3]_19_, [4]_1_, [5]_1_, [6]_32_
7	45000x	223728x	3: [2]_1_, [3]_12_, [6]_12_
8	15616x	26986x	2: [3]_5_, [6]_2_
9	3305x	3305x	1: [3]_1_
10	144x	144x	0: -
11	1x	1x	0: -

Underlined classes belong to an isomorphic SCC that transports a fragment of eight carbon atoms (cf. Fig. 7). Columns "s'up" and "s'up (iso)" give the theoretical speedup factor obtained when running the O(*n*^3^) linear equation solver on the networks decomposed into SCCs and isomorphic SCCs.

**Figure 8:**
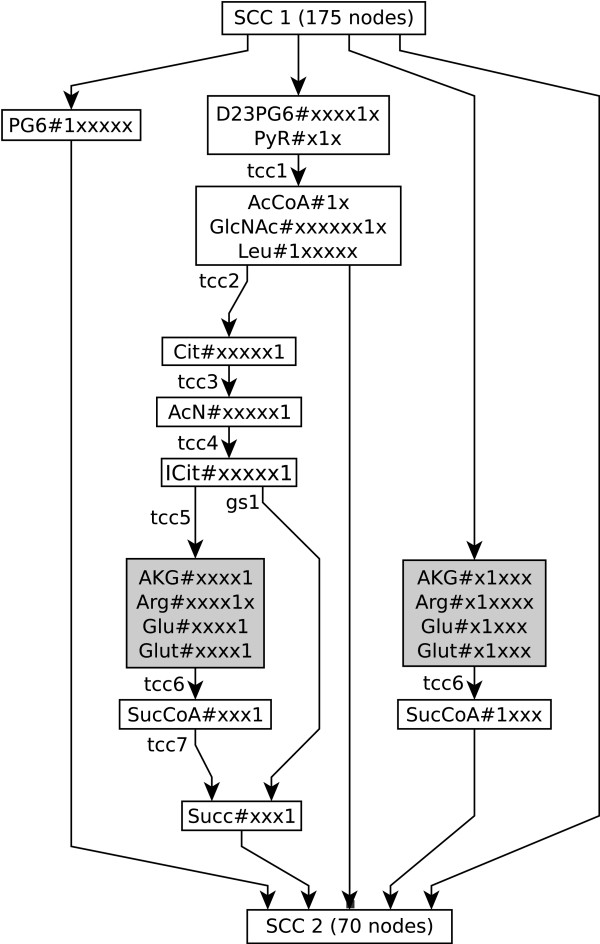
**Component graph of level one**. The component graph (a DAG) of cascade level one in example network "A": two isomorphic SCCs of size four (shaded).

Fig. 8 shows the *component graph *of ^1^*G*. A component graph can be constructed from a digraph by contracting the digraph's SCCs into nodes and removing multiple edges between these new nodes. The component graph is a directed acyclic graph (DAG) by construction. The component graph in Fig. 8 contains two isomorphic SCCs of size four which lie on parallel paths and cannot be reached from each other.

### Global connectivity and reachability

In a reasonable ILN, every labeling position in every pool can be traced back to at least one labeling position of a substrate pool through a path of metabolic reactions. On the other hand, this means that every isotopomer (and therefore also every cumomer) can be synthesized by the network if only the substrate pools consist of a suitably labeled mixture of isotopomers.

Therefore, at least if the fully labeled isotopomer substrate is part of the input substrate mixture, all labeling positions in the network are affected. On the other hand, not all labeling positions of the substrate may be needed to describe the synthesis of a certain isotopomer. Hence, every node of the ILN can be associated with a set of *essential substrate nodes *which fully determines its labeling state.

Furthermore, any node can be associated with a reduced network defined by a set of *essential nodes *which can be found on paths leading from the node's essential substrate nodes to the node itself, i.e. a set of *topological predecessors*. Such a reduced network is typically much smaller than the original network and clearly, a network reduction also yields a dimension reduction of the corresponding IBEs.

#### Path tracing

In general, when tracing paths of isotopes through the metabolic network, there are two distinct perspectives:

1. **forward-tracing **describes how an isotopic labeling of the substrate distributes among the network's pools, i.e. the result of a forward tracing analysis is a the set of nodes affected by a certain labeling of the substrate.

2. **back-tracing **describes the set of nodes feeding a certain node with isotopic labeling, and ultimately the set of input nodes involved in the formation of a metabolite's certain labeling state.

A forward-tracing of the labeling found in the isotopomers of a certain substrate mixture can be performed in the cumomer network e.g. by a depth-first-search starting at the associated substrate cumomers. Special care has to be taken for assembly reactions, i.e. when the depth-first search crosses the levels of the cumomer cascade: the product is reachable from the substrate if and only if all educts are reachable from the substrate. Tab. 6 summarizes some results for the *E. coli *network "A".

**Table 6:** Impact and propagation of a certain isotopomer substrate in example network "A"

substrate isotopomer	affected cumomers	percentage
Glc[1,2,3]_in#100000	10118	98.2%
Glc[1,2,3]_in#010000	10118	98.2%
Glc[1,2,3]_in#001000	10118	98.2%
Glc[1,2,3]_in#000100	138	1.3%
Glc[1,2,3]_in#000010	10118	98.2%
Glc[1,2,3]_in#000001	10118	98.2%
Glc[1,2,3]_in#110000	10124	98.2%
Glc[1,2,3]_in#001100	10124	98.3%
Glc[1,2,3]_in#000011	10214	98.3%
Glc[1,2,3]_in#111000	10136	98.4%
Glc[1,2,3]_in#000111	10136	98.4%
Glc[1,2,3]_in#110011	10160	98.6%
Glc[1,2,3]_in#111100	10160	98.6%
Glc[1,2,3]_in#001111	10160	98.6%
Glc[1,2,3]_in#011111	10208	98.6%
Glc[1,2,3]_in#111111	10304	100.0%

It turns out that single carbon atom positions influence nearly all cumomers in the network (and therefore, also all isotopomers). As expected, the coverage increases with an increasing number of labeled carbon atoms, and, as stated above, the fully labeled substrate isotopomer covers all cumomers in the network. Curiously, the third carbon atom of the glucose substrate influences only a very small number of cumomers.

The back-tracing can be performed by computing the transitive closure of the cumomer cascade. For a selected cumomer, the result of the back-tracing analysis describes a *reduced cumomer network*. Such a reduced network is *minimal *for the cumomer node in question, since it contains only cumomer nodes that are essential for the computation of the selected cumomer's labeling state. In essence, this dimension reduction results in a more efficient solution of LBEs, since only a minimal se of required LBEs has to be solved. For example, Tab. 7 gives the sizes of the reduced networks necessary to describe the isotopomer labeling distributions of the nineteen amino acid pools found in the *E. coli *network "A".

**Table 7:** Reduced networks

pool	atoms	essential cumomer nodes	percentage
Leu	6	297	2.9 %
Gly	2	488	4.7 %
Val	5	649	6.3 %
Arg	6	649	6.3 %
Glu	5	650	6.3 %
Ser	3	652	6.3 %
Ala	3	659	6.4 %
Cys	3	659	6.4 %
Gln	5	681	6.6 %
Pro	5	681	6.6 %
Lys	6	736	7.1 %
Ile	6	739	7.2 %
Met	5	741	7.2 %
Asp	4	742	7.2 %
Thr	4	742	7.2 %
His	6	983	9.5 %
Trp	11	1106	10.7 %
Phe	9	1802	17.5 %
Tyr	9	2313	22.4 %

In case only a small subset of cumomers needs to be simulated, e.g. all cumomers of a single amino acid pool, a *reduced network *can be extracted from the full cumomer network. The reduced network provides a significant dimensional reduction and is minimal in the sense that it consists of the smallest subset of cumomers necessary to describe the desired set of cumomers subject to simulation. As an example the sizes of the reduced networks for all nineteen amino acid pools in the *E. coli *network are shown. Note that if the network size reduces to *p *percent, then also the running time of the simulation algorithm reduces to O(*p*^3^) percent.

Clearly, when forward- or back-tracing hits a node contained in a SCC, immediately all nodes of the SCC are reachable from the substrate nodes and contained in a node's set of essential nodes, respectively. Hence, forward- and back-tracing benefit from the global topological analysis of the cumomer network, since it is much faster to perform this analysis on the component graph than on the unpartitioned network.

By the topology of the cumomer cascade, a cumomer node never depends on a cumomer node of higher weight and therefore the number of a node's topological predecessors naturally increases with increasing weight level of the node. The following statements exemplarily summarize the dependencies for the selected pools GA3P (D-glyceraldehyde 3-phosphate, three carbon atoms) and Lys (L-lysine, six carbon atoms) of the *E. coli *network discussed in sec. *Example network*. This dependency information is generated by evaluating the transitive closure of a graph containing the complete cumomer cascade of network "A", comprised of 10304 cumomer nodes.

• The cumomers of pool GA3P depend on 652 (only 6.3%) other cumomers of the network and only 15 of the 63 cumomers of the three substrate pools Glc1_in, Glc2_in, and Glc3_in are used in the formation of GA3P. Because GA3P consists of only three carbon atoms, this is below the theoretical maximum, since there are 41 substrate cumomers with up to three labeled carbon atoms. Furthermore, GA3P depends on the full sets of cumomers of the following metabolites: AcCoA, CO2, DHAP, FTHF, Gly, GlyOx, m13PGA, m2PGA, m3PGA, PEP, Pyr, Ser.

• The cumomers of pool Lys depend on all cumomers of pool GA3P and additionally on the full set of cumomers of the pools Asp, Fum, Mal, OAA, Succ, SucCoA, and Thr. Although L-lysine has six carbon atoms, it only depends on the same 15 substrate cumomers as GA3P. Because the cumomers of L-lysine depend on 736 other cumomers, a simulation would include only 7.1% of the network's cumomers.

#### General flow of material

The component graph, a DAG that shows the relations between the SCCs of the network, describes the general flow of material through the network. While in level one of the cascade every labeling position in the network is reachable from at least one labeling position of the substrate, this situation changes with increasing fragment size which causes the cumomer network graph to break up into many CCs.

The decomposition of the cumomer network graphs into SCCs also reveals interesting information about how a certain fragment cycles through a sequence of reactions. For example, in Fig. 7 a fragment of eight carbon atoms is transported by a cyclic sequence of four reactions.

Another example is the SCC consisting of 24 nodes found on level one of the cumomer cascade in the unidirectional network "B" (cf. Tab. 4). This SCC describes a cyclic path of a single carbon atom through all reactions of the TCA cycle. Because of the fact, that this is the only SCC describing such a closed path, it can be concluded that only a single carbon atom has the potential to stay on the TCA cycle for one or more rounds. For larger fragments, the TCA cycle is broken up like in the example shown in Fig. 6.

## Conclusion

### Speeding up metabolic flux analysis

Metabolic Flux Analysis (MFA) by using isotopic tracers currently is the most reliable tool for the experimental determination of metabolic fluxes in a living cell. In contrast to more specialized MFA approaches, universal methods for the evaluation of isotope labeling experiments (ILEs) do not rely on special assumptions on network topology, flux reversibility or measurement configuration. One major drawback of universal methods is that they inevitably involve the solution of the full set of nonlinear isotope labeling balance equations (IBEs) describing the flow of labeled material in the network. Moreover, this operation needs to be repeated in a nonlinear parameter fitting procedure for flux estimation.

Consequently, computational complexity can be seen as a major drawback of the inverse simulation method which prohibits its application to larger networks, high throughput experiments or the usage of advanced statistical methods. The present contribution addresses this problem by revealing so far unknown properties of the underlying isotope labeling networks (ILNs) and introducing a novel algorithm for the solution of IBEs by exploiting these properties.

Compared to the classical IBE solution method by cumomer fractions, which can be considered as the state of the art, the new algorithm provides a speed-up by a factor of 1000 for a realistically sized *E. coli *central metabolic example network with about 10^4 ^cumomer nodes (and about 6000 equations to be solved). A prototypical C++ implementation performs a single IBE solution run in only 24 milliseconds. Clearly, by speeding up this central computational step, all further MFA algorithms based on simulation are accelerated by about the same factor.

### Network decomposition

The functional principle of the novel IBE solution algorithm is closely related to the structure of the ILN which describes the isotopic label flow by a directed graph. The computational speed-up is achieved by decomposing the (linear) ILN's weight levels into smaller components. Although this technique for dealing with linear equation systems is well-known from other fields of scientific computing, it strongly depends on the structure of the underlying equations whether this approach is successful. In general, the application domain must have certain physical or geometrical properties that naturally lead to weakly connected or even disconnected network structures.

In fact, the first weight level of ILEs (i.e. the atom transition network) is known to be quite strongly connected, i.e. a network decomposition will only yield a minor speed-up here. This might be the reason why, in this context, network decomposition methods have not been considered before. The new discovery of the present investigation is, that the higher weight level networks tend to break up into a large number of very small components, the strongly connected components (SCCs). Since the networks on upper weight levels are usually of much higher dimension compared to the atom transition network (level one) this explains the dramatic speed-up of equation solution.

The theoretical foundation of ILE network decomposition is given by lemma 1 which, for the first time, rigorously proves the incremental dissolution of weight level networks as an inherent property of ILNs. In particular, the network connectivity decreases monotonously with increasing weight level. This special feature of ILNs is demonstrated for the *E. coli *network where 3716 of 3742 network components have a size smaller than 10.

### Tracing of isotopic labeling

Knowing the fate of labeled molecules in a CLE is an important information for experimental design. Since the 1980ies many researchers have manually traced labeled molecular fragments through ILNs. However, this strategy will never yield a complete structural understanding of label flow. By applying graph theoretic concepts and algorithms such a global understanding of ILNs has been achieved for the first time. In particular, the SCCs reveal all kinds of cyclic flows which is of special interest because these cycles introduce interdependencies between IBEs.

There are two diametral perspectives when tracing isotopic labeling through the network: on the one hand, the *forward-tracing *describes the fate of labeled compounds by identification of the set of affected nodes. This is an especially interesting application for biochemists who are interested in how the metabolic reactions distribute a specific isotopic labeling through the network. On the other hand, the *back-tracing *elucidates the origin of certain fragments, explains their formation and transport through the network. Once a single node of a SCC is involved, all other nodes of the same SCC can be reached, too.

Both, *forward*- and *back-tracing *become particularly simple. Knowing the SCCs the global label flow can be described by an acyclic graph connecting cyclic subnetworks. If one node of a SCCs is involved then all other nodes of the same SCC are reachable by labeled material, too.

As an example for path tracing, the isotopomer distributions of the amino acid pools found in the discussed *E. coli *network were shown to be influenced by only of 5–20% of the nodes found in the original network. When the simulation is allowed to restrict to the metabolic pools that are actually measured, this additionally leads to a considerable dimension reduction. By the application of a similar *back-tracing *scheme a comparable network reduction for mass isotopomer networks was obtained in [39].

### Computational efficiency

Generally, the running time for the solution of a nonlinear equation system, without exploiting any additional structural features, is in the order of ℓ·O(*n*^3^) where *n *is the system's dimension and ℓ the number of iteration steps of a NEWTON-type method. In the case of IBEs the system naturally decomposes into *s *+ 1 weight levels, i.e.

(1) *n *= *n*_0 _+ *n*_1 _+ ... + *n*_*s*_.

Moreover, each level only needs the solution of a linear equation system. This cascaded structure of the IBE equation system was already exploited in [14] and gave rise to a direct numerical solution of complexity

(2) $$\sum_{k=1}^{s}O(n_{k}^{3}).$$

This algorithm can be considered as the present state of the art for IBE solution and is widely applied in the MFA community [39,43,52] and it already yields a significant speed-up of IBE solution. However, the largest *n*_*k *_is typically in the same order of magnitude as *n*, thus dominating the computational effort. For example, in the *E. coli *network max{*n*_*i*_} = *n*_4 _= 1201 ≈ 0.2·*n*.

The global topological analysis now revealed that, particularly, the higher weight level networks with larger values of *n*_*k *_decompose into many smaller subnetworks, i.e.

(3) $$n_{k}=n_{k,1}+n_{k,2}+...+n_{k,t_{k}}.$$

Consequently, the overall computational effort for solving the IBEs reduces to

(4) $$\sum_{k=1}^{s}\sum_{j=1}^{t_{k}}O(n_{k,j}^{3}).$$

Clearly, the precise values of the *n*_*k,j *_depend on the specific network under investigation and can be influenced by different modeling assumptions on reaction reversibilities (c.f. tab. 4). Nevertheless, an approximate upper bound for computing time can be provided, when the size of the largest network component (i.e. max{*n*_*k,j*_}) is known. Because for sufficiently large *ν *it holds

(5) $$m_{1}^{3}+...+m_{\nu }^{3}\ll {\left(m_{1}+...+m_{\nu }\right)}^{3}$$

all network components smaller than max{*n*_*k,j*_} can be rearranged into groups of total size ≈ max{*n*_*k,j*_}. This is possible because (by lemma 1) there will be naturally a majority of very small SCCs. After grouping the SCCs it follows

(6) $$\sum_{k=1}^{s}\sum_{j=1}^{t_{k}}O(n_{k,j}^{3})\ll \frac{n}{\max\{n_{k,j}\}}\cdot O(\max{\left\{n_{k,j}\right\}}^{3}).$$

In practice, this upper bound is very pessimistic as shown with the *E. coli *network. Here, it turned out that max{*n*_*k*, *j*_} = *n*_1, 5 _= 175 ≈ 0.03·*n*, but 99.3% of the SCCs had a size smaller than 10. This explains the achieved speed-up factor compared to the standard method.

### Isomorphic subnetworks

Another important result of the present investigation proven in lemma 3 is the natural occurrence of many isomorphic subnetworks in an ILN. As the lemma suggests, the network is composed of many isomorphic subnetworks which actually lead to classes of identical subsystems sharing the same equation systems, though having different inhomogeneous terms. In fact, the presence of hundreds of isomorphic components could be verified for the *E. coli *network. Here, 2069 of the 2734 components of size > 1 have isomorphic images. This further reduces computational complexity because matrices only need to be factorized once.

Moreover, isomorphism also explains a fact that was known before from an instationary analysis of isotope labeling systems. It turned out empirically by numerical computations that many eigenvalues of these systems occur repeatedly. This multiplicity of eigenvalues is simply caused by classes of isomorphic SCCs. Knowledge on eigenvalues is very important for determining the time constants of an ILE [53].

### Towards genome-wide networks

A great challenge of isotopic MFA is the extension to larger networks up to genome-scale applications. Generally, it is no problem to apply the algorithms for topological analysis to much larger networks with n ≫ 100, 000 nodes because network decomposition is computationally cheap (i.e. O(|^*k*^*V*| + |^*k*^*E*|) ≈ O(*n*)). Moreover, this network analysis procedure needs to be carried out only once. In contrast, the bottleneck is the subsequent solution of the IBEs.

At first glance, the third powers in eq. (4) indicate that genome-scale networks are still out of reach. However, the following consideration reveals that one can be much more optimistic: By eq. (6) the total computing time is dominated by the largest SCC in the network. Moreover, lemma 1 guarantees that this SCC will always lie in network level one (i.e. the carbon atom transition network). If the size of the largest SCC subnetwork is limited the growth of computational effort will only be moderate, even for genome-wide networks.

It follows, that in order to judge the applicability of the new method to a large network only the atom transition network has to be inspected. Clearly, an example network is required to verify these considerations. Fortunately, during the reviewing process of this paper, a first genome-wide metabolic network for *E. coli *was published in [54] which contains not only 184 metabolites and 350 reaction steps but also the corresponding carbon atom transition information for 238 reactions thereof. Although the proposed network cannot be considered to be fully verified at the moment it was prepared for processing with the new IBE solution algorithm. During this process some inconsistencies had to be removed, some corrections were necessary and a few reaction steps have been added. This results in a network containing a total of 169 metabolites and 256 reaction steps. For the sake of brevity the details cannot given here. The metabolic network can be obtained from the authors on request.

**Figure 9:**
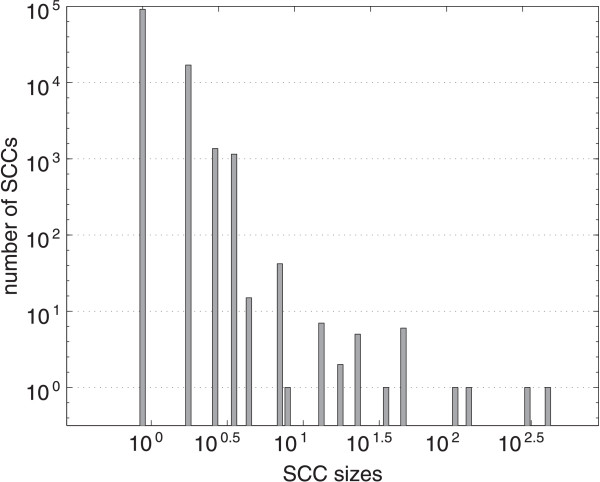
**Histogram of SCC sizes for a genome-wide network**. Logarithmically scaled histogram visualizing frequencies of different SCC sizes of a genome-wide model [54].

This genome-wide network contains 900 carbon atoms (level 1). Because the largest metabolite in the network has 16 carbon atoms the complete network has 136237 cumomer nodes in total. The largest cumomer level (level 7) has 22960 nodes. Fig. 9 shows the SCC statistics of the genome-wide network. The largest SCC on level 1 has dimension 464 which is about three times the largest SCC dimension in the *E. coli *model discussed above. Comprehensive SCCs of the dimensions 340, 140 and 114 where found on different levels; however, 99.98% of the SCCs are of size lower than 10. The number of isomorphic SCCs is growing tremendously. For example there is a large class of 1023 isomorphic SCCs of size four and even six isomorphic SCCs of size 52. The percentage of non-trivial SCCs (i.e. SCCs with size > 1) without isomorphic copies is only 0.04%.

The solution of the linear equation system of level 7 (having in total a size of 22960) using a general O(*n*^3^) method takes in the order of 100 minutes which clearly shows the limitations of the current standard method for repeated IBE solution. In contrast, one single IBE solution step of the genome-wide network, including the decomposition of the 136143 balance equations into CCs and SCCs, can be performed within a computing time of about one second, which suits well for MFA application. Since the largest SCC in the genome-wide network is about three times larger than the one in the smaller *E. coli *network an increase of computing time of about factor 3^3 ^≈ 30 can be expected – indeed an increase about factor 33 is observed. These computing times demonstrate that, by exploiting the topology of ILNs, universal methods for isotopic MFA can now be applied to genome-wide networks.

### General experimental limitations

Because there is currently no way to establish an universal MFA algorithm (in the sense given above) without solving IBE systems any such algorithm will profit from the achieved speedup. This puts the frontier of possible applications further into unknown territory: larger networks, advanced statistical methods and high-throughput evaluations are now within the range of isotope-based MFA methods.

Clearly, the application of the new IBE solution algorithm does not affect the principal applicability of the isotopic flux analysis method because the set of equations that are to be solved remains the same. In particular, the problems of statistical or structural non-identifiability due to insufficient measurement information and of incorrect representation of the biological network persist [55,56]. However, it should be noticed that universal algorithms have less problems here than more specific approaches to MFA which, unlike universal approaches, can never exploit all IBE constraints and the full measurement information.

### Further potential for speeding-up MFA

Recently, an alternate approach to formulate IBEs was introduced in [39]. Hereby, the dimension of ILNs can be dramatically reduced using the concept of elementary metabolite units (EMUs). Noting that this EMU framework relies on exactly the same network structure as treated in the present contribution, any network decomposition will also be beneficial for EMU models. In fact, our method to reduce the computational effort for isotope labeling systems by decomposition into smaller units is *orthogonal *to the EMU approach. This actually means that both approaches – cumomer network decomposition and EMU modeling – can be combined and will certainly yield another speedup which is certainly even higher compared to that of the single methods. Summarizing, network decomposition and EMU modeling are not rivalling approaches but rather synergetic methods. A future contribution covering more numerical details will answer the question how beneficial the combination of both concepts is.

## Abbreviations

CC, Connected Component; CLN, Cumomer Labeling Network; DAG, Directed Acyclic Graph; IBE, Isotopomer Balance Equation; ILE, Isotope Labeling Experiment; ILN, Isotope Labeling Network; LBE, Labeling Balance Equation; MFA, Metabolic Flux Analysis; MNA, Metabolic Network Analysis; MS, Mass Spectrometry; NMR, Nuclear Magnetic Resonance; SCC, Strongly Connected Component

## Authors' contributions

MW developed the methods for the graph theoretical analysis of ILNs including the improved algorithms for forward simulation of labeling networks in isotopically stationary state. KN and WW conceived of the study, participated in its elaboration and coordination and helped to draft the manuscript. All authors read and approved the manuscript.

## Appendix A: Short introduction to isotope-based MFA

### Metabolic Flux Analysis

Metabolic fluxes, or synonymously *in-vivo *reaction rates, represent the operative determinants of cellular function and regulation. Metabolic Flux Analysis (MFA) aims at the precise quantification of all metabolic fluxes in the central metabolic pathways of a microorganism and has become a key technology for Systems Biology. It is an invaluable tool for genetic engineering since the resulting flux map is essential for evaluating the effects of varying growth conditions or genetric manipulations.

Intracellular fluxes are *per se *not directly measurable and have to be estimated from measured quantities through model-based interpretation with the aid of computational routines. Balancing intracellular metabolite pools in a given reaction network and observing extracellular fluxes (uptake and production rates) constitutes the *stoichiometric *MFA approach. In order to determine all unknown fluxes several critical assumptions have to be met which inherently limit the capabilities of this simplest form of flux analysis. Clearly, bidirectional fluxes, metabolic cycles or unknown co-factor utilization are out of reach for stoichiometric MFA [5].

Performing isotope labeling experiments (ILE) makes the labeling state of cellular compounds a measurable and highly informative quantity. Its exploitation can effectively remove the bottleneck of flux determination immanent to stoichiometric MFA. In an ILE, ^13^C-labeled substrate (usually glucose) is fed to the cell in a continuous culture using a chemostat in order to maintain metabolic stationarity. Driven by the highly specific enzymatic reactions, the ^13^C isotopes distribute among the cell's metabolites until the isotope labeling becomes equilibrate throughout the network. In the classical, stationary ^13^C MFA the metabolic intermediates are sampled. The metabolites' emerging specific isotope isomer (isotopomer) patterns can be measured using mass spectrometry (MS, [57]) or nuclear magnetic resonance (NMR, [17]) methods.

### Computational procedure

**Figure 10:**
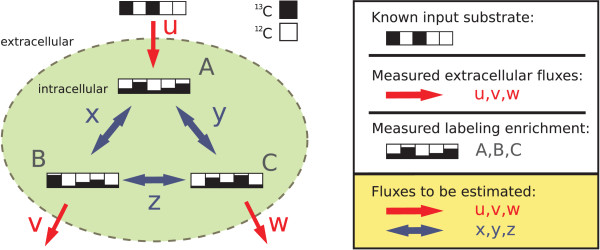
**Principle of isotope-based MFA**. Labeled input substrate, extracellular fluxes and intracellular labeling patterns are used to determine the intracellular fluxes in-vivo.

In order to reconstruct the unknown fluxes from the measured data a complex mathematical modeling and computational simulation procedure has to be applied. Accepting as basic assumptions that the metabolites are homogeneously distributed and no mass isotope effects occur, the general computational sequence shall be illustrated here with the example network shown in Fig. 2 which is already discussed in the main text. Despite of its simplicity, this toy example is sufficient to outline the main aspects here. For an introduction into the general modeling framework of classical, stationary ^13^C ILEs reader is referred to [12,14]. In the classical, stationary 13C MFA the metabolic intermediates are sampled, (cf. Fig. 10).

#### Step 1: Stoichiometric equations

As a preliminary, the model of the metabolic network under investigation has to be set up. Following the law of mass conservation, stoichiometric equations of the stationary reaction system can be formulated [58,59] without including any labeling information. One balance equation has to be specified for each intermediate metabolite pool in the system, i.e. the sum of all fluxes arriving in a pool equals the sum of the fluxes that leave it (cf. Fig. 2a, where now *v*_*i *_denotes the flux value of the edge *σ*_*i*_, *i *= 0,...,4):

(7) $$\begin{array}{cccc} pool & influx & & efflux\\ \text{A}: & v_{0}+v_{3} & = & v_{1}\\ \text{B}: & v_{1} & = & v_{2}+v_{4}\\ \text{C},\text{D}: & v_{2} & = & v_{3} \end{array}$$

#### Step 2: Flux constraints

Eqns. (7) constitute four equations for five fluxes. Due to the redundancy of the equations for pools C and D this leaves two degrees of freedom for the system solution, i.e. if two of the fluxes are known all other fluxes can immediately be calculated. Commonly, the uptake flux *v*_0 _is assumed to be measurable. If then, for instance, *v*_2 _is fixed, the cycle is determined. The fluxes *v*_0_, *v*_2 _are called *free fluxes*, while all other fluxes are called *dependent*. Notice, that the choice of free fluxes is usually neither unique (e.g. instead of *v*_2_, both *v*_1 _or *v*_3 _are feasible choices) nor is any set feasible (e.g. *v*_0 _and *v*_4_). By appointing the free fluxes which are subject to the parameter fitting procedure (cf. Step 5) a parameterization of the stoichiometric model is achieved. Thus, in order to determine the free fluxes at least some additional information is required that can be supplied by CLEs.

#### Step 3: Simulation of a CLE

The required structural information for tracing isotopic labeling through the atom backbone of the metabolic pathways is described by the atom network (cf. Fig. 2b). For realistic networks this information nowadays can be taken from biochemistry textbooks. Based on this representation the isotopomer labeling network (ILN) is derived (cf. Fig. 2c) which explains the formation of every isotopomer in the reaction system.

As starting point for the computation of synthetic labeling distribution arising from a specifically labeled input substrate, mass balance equations are derived for every isotopomer fraction in the ILN. For instance, three of the twelve isotopomer balance equations(IBEs) for the network in Fig. 2c are:

(8)

The first equation reveals a fundamental problem of IBEs: assembly reactions introduce nonlinear terms into the equations (*v*_3_•(C○)•(D•) in this case). Besides the large number of IBEs, say *n*, their nonlinearity is the major reason why the running time of basically all numerical methods used for their solution is at least O(*n*^3^).

Applying the translation rules given in Def. 2 the IBEs (8) are transformed into a set of cumomer balance equations (CBEs) having cumomer fractions as unknowns. Interestingly it turns out that CBEs can be partitioned by *weight *(the number of labeled (•) positions, cf. Def. 1).

Here, the CBEs which correspond to cumomers with an  label in all positions (weight 0) are redundant because the value of the associated cumomer fractions is always 1. Hence, they reduce to the stoichiometric equations (7). For the network shown in Fig. 2c this results in the modified network shown in Fig. 3 and then following eight CBEs:

(9)

As a main difference to IBEs, there are no splitting reactions in a cumomer network, in the sense that a cumomer distributes its labeled positions to more than one product. The equation for  shows that the transformation into cumomers did not remove all nonlinear terms. If, however, the subnetworks defined by the cumomers' weight are solved consecutively in the order of ascending weight the nonlinear terms can be easily evaluated because they appear as constants in the subsequent equations. This cascaded structure of the CBEs makes them accessible for accurate and efficient direct solution methods (i.e. linear equation solvers) when both the free fluxes and the input specification of E are given. The calculation step towards the stationary cumomer/isotopomer state is referred to as *forward *simulation problem. The obtained solutions, cumomer fractions, can be easily converted back into isotopomer fractions by a linear transformation.

#### Step 4: Modeling of Measurements

To actually evaluate the ILE one operative step is still missing: the relation between the isotopomer/cumomer fractions of a metabolite pool and their quantitative labeling measurements. Accordingly, the example is now extended by a measurement scenario to illustrate the modeling process. To this end, the metabolite B is exemplary assumed to be observable by the use of MS technique. Analogous equations can be derived for other measurement methods like ^1^H- or ^13^C-NMR [26]. In case of MS, molecules with 0, 1 or 2 labeled carbon atoms are distinguishable. Thus, ideally three mass isotopomer fractions can be observed. After correcting the mass shift due to naturally occurring stable isotopes [52] the associated measurement equations read:

(10)

Here, the samples *y*_*b*, *i*_, *i *= 1, 2, 3 are affected by measurement errors *ε*_*i *_which are typically assumed to be independent and normally distributed with Zero mean and standard deviations *σ*_*i*_. These quantities are estimated by repetition of experiments or expert knowledge about the overall sampling and analytic procedure. Notice that one of the three values *y*_*b*, *i*_, *i *= 1, 2, 3 is redundant with the other two because their sum must always be one.

It turns out that the measurement information (10) alone is not capable of determining all the isotopomer fractions of B. At the utmost, sophisticated combination of several data sets from different analytic methodologies can provide that. Clearly, the ratio between the number of available MS measurements and from such data identifiable isotopomers becomes worse when the number of carbon atoms increases.

#### Step 5: Flux estimation

**Figure 11:**
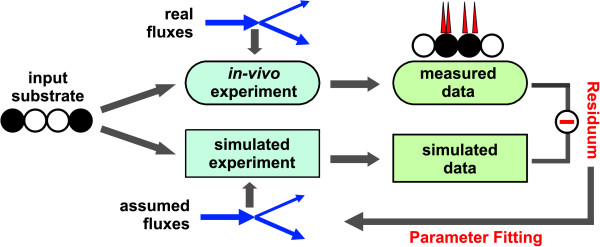
**Workflow of an isotope-based MFA**. After the evaluation of a real ILE measurement data is available for the mathematical modeling. Initially starting with a guess on the flux values, a simulation of the ILE aims to reproduce the measurement data. A parameter fitting procedure is used to obtain an estimation of the real flux values by gradual variation.

Relying on the Steps 1–4 a mathematical model for flux estimation is composed which describes the relation between free fluxes and measured data. Typically, the flux distribution is identified by fitting the fluxes to the observed data, whereby the difference between simulated and measured isotope labeling patterns is minimized in an iterative procedure [13], Figure 11.

The general optimization problem is given by minimizing the sum *S *of residuals subject to the free fluxes:

(11) $$\left({\hat{v}}_{0},{\hat{v}}_{2})=\arg\underset{v_{0},v_{2}}{\min}S(v_{0},v_{2}\right)$$

In order to mimic the varying precision of the data, each residual is weighted with the inverse variance of the associated measurement error. For the running example the weighted sum of squares reads:

(12)

where $v_{0}^{\text{meas}}$ is the observed uptake rate with variance $\sigma_{v_{0}}$ and the arguments are left out for convenience. Application of state of the art optimization routines (of global character or based on local optimization connected with an appropriate *multistart strategy*) in an iterative procedure then provides the desired flux estimates ${\hat{v}}_{0}$, ${\hat{v}}_{2}$.

The question whether the measured information is sufficient to determine the unknown free fluxes is referred to as identifiability analysis and requires sophisticated statistical methods. E.g., data consistency checking can be done by performing a *χ*^2 ^test. It is not aimed to assess these advanced topics here. For details the reader is referred to [15]

## Appendix B: Proofs

### Proof of lemma 1

Lemma 1 states that the connectivity of ^*k*^*G*, i.e. *ε*(^*k*^*G*) = |^*k*^*E*|/|^*k*^*V*|, decreases monotonically with increasing weight level *k*, i.e. *ε*(^*k*+1^*G*) ≤ *ε*(^*k*^*G*). The lemma can be justified by a counting argument:

#### Proof

The fixed number of cumomer nodes of a metabolite on weight level *k *is given by the number of combinations, i.e. $\left(\begin{array}{c} s\\ k \end{array}\right)$, where *s *is the number of available labeling positions in the metabolite. Obviously, only metabolites with at least *k *labeling positions contribute cumomers to weight level *k*.

• For unimolecular reactions of type A → B, pools A and B necessarily have the same number of labeling positions. The number of edges linking the cumomers of both pools equals the number of cumomers of pool A or B, since pool B is obtained from A by a permutation of labeling positions. Hence, the number of edges is proportional to the number of nodes by a constant a throughout the levels k of the cascade, i.e. |^*k*^*E*| = *α*·|^*k*^*V*|, and consequently *ε*(^*k*+1^*G*) = *ε*(^*k*^*G*).

• For any bimolecular reaction A + B ⇌ C where molecules A and B have *s*_*a *_and *s*_*b *_labeling positions, and pool C has *s*_*a *_+ *s*_*b *_labeling positions, there exist weight conserving cumomer transitions A ⇌ C, B ⇌ C. Since the inequality $\left(\begin{array}{c} s_{a}\\ k \end{array}\right)+\left(\begin{array}{c} s_{b}\\ k \end{array}\right)\leq \left(\begin{array}{c} s_{a}+s_{b}\\ k \end{array}\right)$ holds (which follows from VANDERMONDE's *Convolution*; see e.g. [60]), the number of cumomers of pool Con level *k *of the cascade is always greater than or equal the number of cumomers of pools A and B. Hence, with increasing level *k*, there exist cumomers of pool C which have no reaction partners in pools A and B and the number of cumomers grows faster than the number of transitions, i.e. *ε*(^*k*+1^*G*) ≤ *ε*(^*k*^*G*).   □

### Proof of lemma 3

Lemma 3 states that a sequence of reactions transporting a maximal fragment consisting of *s *labeling positions induces $\left(\begin{array}{c} s\\ k \end{array}\right)$ disjoint isomorphic paths on weight level *k *of the cascade:

#### Proof

Because *width *($R_{1},...,R_{\ell }$, P_0_,..., P_ℓ_) = *s *the reaction sequence transports a fragment of size *s*. It follows, that there are $\left(\begin{array}{c} s\\ k \end{array}\right)$ isomorphic paths on weight level *k *of the cascade and $\sum_{k=1}^{s}\left(\begin{array}{c} s\\ k \end{array}\right)=2^{s}-1$ isomorphic paths on weight levels 1,..., *s*.

Furthermore, there are no isomorphic paths beyond weight level *s*: if the largest participating atom backbone has more than *s *atoms then at least one of the atom backbones of size *s *is the result of a splitting reaction. The split off atoms must not be labeled otherwise the weight-conservation constraint would be violated (rule 2 of Def. 2). For the same reason, assembly reactions may only append unlabeled atoms, because any labeled atom would increase the weight.

All isomorphic paths on weight level *k *are guaranteed to be disjoint, i.e. they do not share common cumomer nodes, since reactions $R_{1},...,R_{\ell }$ are always one-one mappings (permutations) of labeling positions.   □

## Supplementary Material

Additional file 1 Reactions and carbon atom transitions of the *E. coli *example network. The list contains the subset of metabolic reactions considered for the *E. coli *example network shown in Fig. 5. The letters following the "#" symbol denote the metabolite's carbon atoms and the specific transfer of carbon atoms performed by an enzyme [42]. Only the reactions shown in Tab. 2 are assumed to be unidirectional. Click here for file
