# Supplementary material for: The topology of metabolic isotope labeling networks
Source: BMC Bioinformatics. 2007 Aug 29;8:315. doi: 10.1186/1471-2105-8-315 (PMC2233644; doi:10.1186/1471-2105-8-315)
Supplement: Additional file 1 — Reactions and carbon atom transitions of the E. coli example network. The list contains the subset of metabolic reactions considered for the E. coli example network shown in Fig. 5. The letters following the "#" symbol denote the metabolite's carbon atoms and the specific transfer of carbon atoms performed by an enzyme [42]. Only the reactions shown in Tab. 2 are assumed to be unidirectional. [file 1471-2105-8-315-S1.pdf]

Additional File 1:

Reactions and carbon atom transitions of the E. coli example network

The following list contains the subset of metabolic reactions considered for the *E. coli* example network shown in Fig. 5. The letters following the "#" symbol denote the metabolite's carbon atoms and the specific transfer of carbon atoms performed by an enzyme [1]. Only the reactions shown in Tab. 2 are assumed to be unidirectional.

i_Glc1:	Glc1_in#ABCDEF	⇌	Glc6P#ABCDEF
i_Glc2:	Glc2_in#ABCDEF	⇌	Glc6P#ABCDEF
i_Glc3:	Glc3_in#ABCDEF	⇌	Glc6P#ABCDEF
o_AcCoA:	AcCoA#AB	⇌	O_AcCoA#AB
o_ace:	AcCoA#AB	⇌	Ace#AB
o_Asp:	Asp#ABCD	⇌	O_Asp#ABCD
o_bs_akg4:	Arg#ABCDEF	⇌	O_Arg#ABCDEF
o_bs_fru6P:	GlcNAc#ABCDEFGH	⇌	O_GlcNAc#ABCDEFGH
o_bs_oaa2:	Met#ABCDE	⇌	O_Met#ABCDE
o_bs_oaa4:	Ile#ABCDEF	⇌	O_Ile#ABCDEF
o_bs_oaa56:	Lys#ABCDEF	⇌	O_Lys#ABCDEF
o_bs_pep3:	Phe#ABCEFGHIJ	⇌	O_Phe#ABCEFGHIJ
o_bs_pep4:	Tyr#ABCEFGHIJ	⇌	O_Tyr#ABCEFGHIJ
o_bs_pep5:	Trp#ABCDEFGHIJK	⇌	O_Trp#ABCDEFGHIJK
o_bs_pga3:	Gly#AB	⇌	O_Gly#AB
o_bs_pyr2:	Val#ABCDE	⇌	O_Val#ABCDE
o_bs_pyr4:	Leu#ABCDEF	⇌	O_Leu#ABCDEF
o_bs_rib5p1:	His#ABCDEF	⇌	O_His#ABCDEF
o_CO2:	CO2#A	⇌	O_CO2#A
o_Ery4P:	Ery4P#ABCD	⇌	O_Ery4P#ABCD
o_Fru6P:	Fru6P#ABCDEF	⇌	O_Fru6P#ABCDEF
o_Glc6P:	Glc6P#ABCDEF	⇌	O_Glc6P#ABCDEF
o_Glut:	Glut#ABCDE	⇌	O_Glut#ABCDE
o_Glyc:	DHAP#ABC	⇌	O_Glyc#ABC
o_P5P:	RNA#ABCDE	⇌	O_P5P#ABCDE
o_PEP:	PEP#ABC	⇌	O_PEP#ABC
o_Pyr:	Pyr#ABC	⇌	O_Pyr#ABC
ana1:	PEP#ABC + CO2#a	→	OAA#ABCa

ana2:	Mal#ABCD	$\rightarrow$	Pyr#ABC + CO2#D
bs_akg1:	AKG#ABCDE	$\rightleftharpoons$	Glu#ABCDE
bs_akg2:	Glu#ABCDE	$\rightleftharpoons$	Pro#ABCDE
bs_akg3:	Glu#ABCDE	$\rightleftharpoons$	Gln#ABCDE
bs_akg4:	Glu#ABCDE + CO2#a	$\rightleftharpoons$	Arg#ABCDEa
bs_akg:	AKG#ABCDE	$\rightleftharpoons$	Glut#ABCDE
bs_fru6P:	Fru6P#ABCDEF + AcCoA#ab	$\rightleftharpoons$	GlcNAc#ABCDEFab
bs_glc6P:	Glc6P#ABCDEF	$\rightleftharpoons$	Tre#ABCDEF
bs_oaa1:	OAA#ABCD	$\rightleftharpoons$	Asp#ABCD
bs_oaa2:	OAA#ABCD + FTHF#a	$\rightleftharpoons$	Met#ABCDa
bs_oaa3:	Asp#ABCD	$\rightleftharpoons$	Thr#ABCD
bs_oaa4:	Thr#ABCD + Pyr#abc	$\rightleftharpoons$	Ile#ABbCDc + CO2#a
bs_oaa6a:	OAA#ABCD + Pyr#abc	$\rightleftharpoons$	Lys#ABCDcb + CO2#a
bs_oaa6b:	OAA#ABCD + Pyr#abc	$\rightleftharpoons$	Lys#abcDCB + CO2#A
bs_oaa:	OAA#ABCD	$\rightleftharpoons$	Asp#ABCD
bs_pep1:	PEP#ABC + Ery4P#abcd	$\rightleftharpoons$	DAHP#ABCabcd
bs_pep2:	PEP#ABC + DAHP#abcdefg	$\rightleftharpoons$	Chor#ABCabcdefg
bs_pep3a:	Chor#ABCDEFGHIJ	$\rightleftharpoons$	Phe#ABCEFGHIJ + CO2#D
bs_pep3b:	Chor#ABCDEFGHIJ	$\rightleftharpoons$	Phe#ABCEJIHGF + CO2#D
bs_pep4a:	Chor#ABCDEFGHIJ	$\rightleftharpoons$	Tyr#ABCEFGHIJ + CO2#D
bs_pep4b:	Chor#ABCDEFGHIJ	$\rightleftharpoons$	Tyr#ABCEJIHGF + CO2#D
bs_pep5:	Chor#ABCDEFGHIJ + Rib5P#abcde	$\rightleftharpoons$	Trp#edcbaJEFGHI + PyrCO2#ABCD
bs_pep6:	PyrCO2#ABCD	$\rightleftharpoons$	Pyr#ABC + CO2#D
bs_pga1:	m3PGA#ABC	$\rightleftharpoons$	Ser#ABC
bs_pga2:	Ser#ABC	$\rightleftharpoons$	Cys#ABC
bs_pga3:	Ser#ABC	$\rightleftharpoons$	Gly#AB + FTHF#C
bs_pyr1:	Pyr#ABC	$\rightleftharpoons$	Ala#ABC
bs_pyr2:	Pyr#ABC + Pyr#abc	$\rightleftharpoons$	Val#ABbcC + CO2#a
bs_pyr3:	Pyr#ABC + Pyr#abc	$\rightleftharpoons$	AKV#ABbcC + CO2#a
bs_pyr4:	AKV#ABCDE + AcCoA#ab	$\rightleftharpoons$	Leu#abBCDE + CO2#A
bs_rib5p1:	Rib5P#ABCDE + FTHF#a	$\rightleftharpoons$	His#EDCBAa
bs_rib5p2:	Rib5P#ABCDE	$\rightleftharpoons$	DNA#ABCDE
bs_rib5p3:	Rib5P#ABCDE	$\rightleftharpoons$	RNA#ABCDE
edp1:	Glc6P#ABCDEF	$\rightleftharpoons$	DGL6P#ABCDEF
edp2:	DGL6P#ABCDEF	$\rightarrow$	PG6#ABCDEF
edp3:	PG6#ABCDEF	$\rightarrow$	D23PG6#ABCDEF
edp4:	D23PG6#ABCDEF	$\rightleftharpoons$	GA3P#ABC + Pyr#DEF
emp1:	Glc6P#ABCDEF	$\rightleftharpoons$	Fru6P#ABCDEF
emp2:	Fru6P#ABCDEF	$\rightarrow$	FruBP#ABCDEF

emp3:	FruBP#ABCDEF	⇌	DHAP#ABC + GA3P#DEF
emp4:	DHAP#ABC	⇌	GA3P#CBA
emp5:	GA3P#ABC	⇌	m13PGA#ABC
emp6:	m13PGA#ABC	⇌	m3PGA#ABC
emp7:	m3PGA#ABC	⇌	m2PGA#ABC
emp8:	m2PGA#ABC	⇌	PEP#ABC
emp9:	PEP#ABC	→	Pyr#ABC
gs1:	ICit#ABCDEF	→	GlyOx#AB + Succ#DCEF
gs2:	GlyOx#AB + AcCoA#ab	→	Mal#ABba
ppp1:	PG6#ABCDEF	→	CO2#A + Rul5P#BCDEF
ppp2:	Rul5P#ABCDE	⇌	Xyl5P#ABCDE
ppp3:	Rul5P#ABCDE	⇌	Rib5P#ABCDE
ppp4:	Xyl5P#ABCDE + Ery4P#abcd	⇌	GA3P#CDE + Fru6P#ABabcd
ppp5:	Xyl5P#ABCDE + Rib5P#abcde	⇌	Sed7P#ABabcde + GA3P#CDE
ppp6:	GA3P#ABC + Sed7P#abcdefg	⇌	Ery4P#defg + Fru6P#abcABC
ppp_P5P:	Rul5P#ABCDE	⇌	RNA#ABCDE
tcc1:	Pyr#ABC	→	AcCoA#BC + CO2#A
tcc2:	OAA#ABCD + AcCoA#ab	→	Cit#DCBAba
tcc3:	Cit#ABCDEF	→	AcN#ABCDEF
tcc4:	AcN#ABCDEF	→	ICit#ABCDEF
tcc5:	ICit#ABCDEF	→	AKG#ABCEF + CO2#D
tcc6:	AKG#ABCDE	→	SucCoA#BCDE + CO2#A
tcc7:	SucCoA#ABCD	→	Succ#ABCD
tcc8a:	Succ#ABCD	→	Fum#ABCD
tcc8b:	Succ#ABCD	→	Fum#DCBA
tcc9:	Fum#ABCD	→	Mal#DCBA
tcc10:	Mal#ABCD	⇌	OAA#ABCD

References

[1] Wiechert W, Wurzel M: **Metabolic isotopomer labeling systems. Part I: global dynamic behavior**. *Mathematical Biosciences* 2001, **169**:173–205.
